# *Hemerocallis citrina* Baroni leaf total phenol alleviates depressive-like behaviors via modulating “microbiota-gut-brain” axis in chronic unpredictable mild stress -induced rats

**DOI:** 10.3389/fphar.2025.1642515

**Published:** 2025-09-08

**Authors:** Yanping Wang, Yanjun Jia, Wanning Zhao, Yazhou Shao, Zeyu Zhang, Huiru Chen, Shuangxi Qian, Fangdi Hu

**Affiliations:** ^1^School of Basic Medical Sciences, Lanzhou University, Lanzhou, China; ^2^School of Pharmacy, Lanzhou University, Lanzhou, China; ^3^ Gansu Codonopsis Industrial Technology Engineering Research Center, Lanzhou, China; ^4^ Lanzhou Foci Pharmaceutical Co., LTD., Lanzhou, China

**Keywords:** Hemerocallis citrina Baroni leaf, total phenols, chronic unpredictable mild stress, microbiota-gut-brain axis, antidepressant activity, gut microbiota, non-targeted metabolomics

## Abstract

**Background:**

Depression is closely associated with dysfunction of the microbiota-gut-brain axis (MGBA). Total phenol from *Hemerocallis citrina* leaves (HLTP) exhibits antidepressant potential, but its role in relieving depression by regulating MGBA remains uninvestigated.

**Methods:**

In this study, the chronic unpredictable mild stress (CUMS) method was used to establish a rat model of depression to investigate the ameliorative effects of HLTP on depression.

**Results:**

Behavioral and pathological results showed HLTP improved weight loss, increased sucrose preference, reduced forced swimming immobility time, novel-suppressed feeding latency, open field movement trajectory, and alleviated hippocampal CA1 neuronal damage in CUMS-depressed rats. At 10, 20, and 40 mg/kg, HLTP increased dopamine (DA), noradrenaline (NE), 5-hydroxytryptamine (5-HT), interleukin-10 (IL-10), brain-derived neurotrophic factor (BDNF), and cAMP response element-binding protein (CREB) in the brain. It also decreased the inflammatory factor (TNF-α and IL-1β) in the brain, as well as corticotropin-releasing hormone (CRH), adrenocorticotropic hormone (ACTH), and corticosterone (CORT) in serum and brain. HLTP modulated gut microbiota diversity and reversed CUMS-induced alterations in 15 bacterial taxa, including *Alloprevotella* and *Monoglobus*. Additionally, HLTP reversed 63 CUMS-induced abnormal serum metabolites, primarily affecting tryptophan metabolism, niacin and nicotinamide metabolism, and porphyrin metabolism. Significant correlations were observed among gut microbial composition, serum metabolite levels, biochemical indices, and behavioral outcomes.

**Conclusion:**

HLTP exerts antidepressant effects in CUMS-induced rats by modulating the MGBA. It enhances metabolic function, reduces neuroinflammatory damage, restores neurotrophic factor levels, suppresses hyperactivity of the hypothalamic-pituitary-adrenal axis, and corrects monoamine neurotransmitter deficiencies, collectively contributing to the attenuation of depression-related symptoms.

## Graphical Abstract

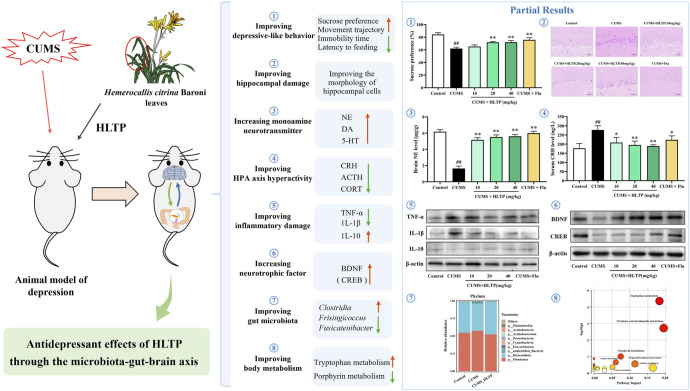

## 1 Introduction

Depression constitutes a psychiatric disorder marked by high prevalence, recurrence, disability, and mortality rates (Jin et al., 2019). It is estimated that 5% of adults worldwide suffer from depression each year, with the highest incidence rate among young people (Herrman et al., 2022), rsulting in substantial societal and familial burdens. The pathophysiology of depression is thought to be multifactorial, involving several key hypotheses: the monoamine deficiency hypothesis, oxidative stress hypothesis, neurotrophic factor deficiency hypothesis, proinflammatory cytokine hypothesis, HPA axis negative feedback dysregulation hypothesis, and gut microbiota dysbiosis hypothesis. Recently, an expanding array of scientific reports has highlighted the crucial role of the microbiota-gut-brain axis (MGBA) in the pathogenesis of neuropsychiatric disorders, particularly depression (Foster and McVey Neufeld, 2013). A “channel” that allows for two-way communication between the gastrointestinal tract and the central nervous system is called the MGBA. This dynamic signaling system consists of numerous components, including the immune system, neuroimmune system, autonomic nervous system, enteroendocrine system, enteric nervous system, hypothalamic-pituitary-adrenal (HPA) axis, and various neurotransmitters (Cryan et al., 2019). Therefore, the MGBA is not a simple linear pathway, but rather a highly integrated and complex regulatory network composed of neural, endocrine, and immune interactions (Agirman and Hsiao, 2021).

*Hemerocallis citrina* Baroni (*H. citrina*), a plant of the genus *Hemerocallis* in the *Asphodelaceae* family, is traditionally used for its medicinal flower buds. In the *Compendium of Materia Medica,* “Xuancao” (daylily) is described as an herb that “regulates the chest and diaphragm, harmonizes the five viscera, induces happiness, and dispels sorrow”, earning it the name “Forgetting Sadness Grass” (Zhang et al., 2004). *H. citrina* has a long history of cultivation in China and has been widely utilized for both dietary and medicinal applications across millennia. Modern pharmacological studies have demonstrated its broad range of bioactivities, including antidepressant (Liu et al., 2022), antioxidant (Zhang et al., 2004), sleep-promoting (Uezu, 1998), and anticancer properties (Cichewicz et al., 2004). Notably, the ethanol extract of *H. citrina* has shown significant antidepressant activity (Yi et al., 2012), and its major phenolic constituents—quercetin (Rinwa and Kumar, 2013), quercetin 3-O-α-L-arabinoside (Shen et al., 2019), rutin (Ebokaiwe et al., 2023), and hyperoside (Zheng et al., 2012)—have been individually confirmed to exert antidepressant effects. In mainland China, *H. citrina* leaves are commonly discarded as agricultural waste. In contrast, in Taiwan Province, dried leaves are segmented and sold commercially as sleep-aid products. However, to date, no pharmacodynamic investigations of *H. citrina* leaves have been reported. This gap significantly limits the potential of *H. citrina* leaves as a raw material for food and medicine, and hinders its effective utilization in food and medicine development, potentially leading to unnecessary waste of resources. Recently, our research group found that the total phenolic content in the ethanol extract of *H. citrina* leaves reaches 1.8 times the concentration observed in the flower extract. We identified 32 phenolic compounds in the total leaf phenolic fraction (HLTP), including quercetin, quercetin 3-O-α-L-arabinoside, rutin, and hyperoside—components also present in the flowers. In addition, phenolic acids derived from caffeoyl and feruloyl groups—such as 4-O-p-coumaroylquinic acid, 4-O-caffeoylquinic acid, 5-O-feruloylquinic acid, and 5-O-caffeoylshikimic acid—were detected in the leaf extract and have exhibited neuroprotective effects *in vitro* (Jia et al., 2024), suggesting potential antidepressant activity. Based on the reported antidepressant effects of these shared components and our previous findings demonstrating the neuroprotective properties of HLTP, we hypothesize that HLTP may exert antidepressant effects.

HLTP’s main components, quercetin and quercetin 3-O-α-L-arabinopyranoside, have been shown to have antidepressant effects in depression models caused by chronic unpredictable mild stress (CUMS). Through upregulating nuclear factor E2-related factor 2 (Nrf2), inhibiting the expression of inducible nitric oxide synthase (iNOS) in the hippocampus, and activating the PI3K/Akt/Nrf2/HO-1 signaling pathway, quercetin reduces the depressive behaviors brought on by CUMS (Guan et al., 2021). Meanwhile, quercetin 3-O-α-L-arabinopyranoside attenuates CUMS-induced neuroinflammation and hippocampal apoptosis by inhibiting the MEK/ERK/NF-κB signaling pathway, thereby exerting antidepressant effects (Shen et al., 2019). An increasing body of evidence from CUMS-based preclinical models has confirmed the close association between depression and the MGBA. It is hypothesized that HLTP may mitigate depressive-like behavior induced by CUMS by regulating the MGBA, in light of these observations. So, to find out how HLTP could alleviate depression through mechanisms mediated by the MGBA, this study used a rodent model of chronic unpredictable mild stress.

## 2 Materials and methods

### 2.1 Drugs and reagents

Gansu Province, China’s Qingyang City is where *Hemerocallis citrina* Baroni leaves were gathered. Chief Pharmacist Xicang Yang of Gansu University of Chinese Medicine’s Affiliated Hospital verified that the plant material was the leaves of *Hemerocallis citrina* Baroni (Asphodelaceae).

High-efficiency RIPA lysis buffer (R0010) was purchased from Cytiva. Norepinephrine (NE, SN8550), dopamine (DA, SD8600), and the SDS-PAGE gel preparation kit (P1200) were obtained from Solarbio. ECL chemiluminescent substrate (GK1008) was purchased from GLPBIO. Antibodies against β-actin (YM3028), tumor necrosis factor-α (TNF-α, YT4689), interleukin-1β (IL-1β, YM4682), interleukin-10 (IL-10, YT5138), brain-derived neurotrophic factor (BDNF, YT7890), and cAMP response element-binding protein (CREB, YM4167) from were supplied by ImmunoWay; Enzyme-linked immunosorbent assay (ELISA) kits for corticotropin-releasing hormone (CRH, MM-0786R1), adrenocorticotropic hormone (ACTH, MM-0565R1), and corticosterone (CORT, MM-0559R1) were purchased from MEIMIAN. A 4% paraformaldehyde solution (BL539A) was obtained from Biosharp. 5-hydroxytryptamine (5-HT, CAS 50-67-9) was provided by Aladdin, and fluoxetine hydrochloride (CAS 56296-78-7) was purchased from Macklin.

### 2.2 Preparation of total phenols from H. citrina leaves

The preparation of HLTP was conducted according to the procedure outlined in our prior work (Jia et al., 2024). Briefly, powdered leaves of *H. citrina* were subjected to reflux extraction using 80% ethanol. The resulting extract was concentrated into a crude paste, which was then redissolved in water and applied to a DM-21 macroporous adsorption resin column for phenolic enrichment. The eluate was subsequently concentrated and lyophilized to yield HLTP. Compound identification in HLTP was carried out using ultra-performance liquid chromatography combined with quadrupole time-of-flight tandem mass spectrometry (UPLC-Q-TOF-MS/MS). The total ion chromatogram of HLTP is presented in Supplementary Figure S1, and detailed structural information on the 32 identified compounds is available in our previous publication (Jia et al., 2024).

### 2.3 Establishment of CUMS rat model and animal grouping

Sixty SPF-grade male Sprague-Dawley (SD) rats (weighing 180–220 g) were obtained from the Experimental Animal Center of Lanzhou University [License No.: SCXK(Gan)-20230003]. All laboratory procedures were carried out in accordance with World Medical Association ethical guidelines, and the Ethics Committee of Lanzhou University’s School of Pharmacy approved them. The animals were housed in standardized laboratory conditions, including a 12-h light/dark cycle, controlled temperature (22°C–25°C), relative humidity (55% ± 10%), and free access to standard chow and water. After a 1-week adaptation phase, official testing commenced.

The CUMS model, known to simulate endogenous depressive states by inducing behavioral abnormalities and elevated plasma cortisol levels, is commonly employed in antidepressant screening and studies on the pathophysiology of depression (Gómez et al., 1999). The CUMS procedure was adapted from previously reported protocols (Zeng et al., 2022), with minor modifications. One stressor was randomly applied each day from the following list: (A) 24 h food deprivation; (B) 24 h water deprivation; (C) Tail clamping with a wooden clip (2 min); (D) Cage tilting at 45° for 24 h; (E) Forced swimming in 4°C water (5 min); (F) Circadian rhythm inversion; (G) Damp bedding for 24 h; (H) Restraint stress for 1 h. To minimize stress adaptation, no stressor was repeated on consecutive days. The modeling period lasted for 35 days, with procedures performed consistently throughout. Body weights were recorded before the initiation of modeling and subsequently measured weekly at 9:00 a.m.

Sixty SD rats were randomly assigned to six groups based on body weight: Normal group (distilled water, 10 mL/kg), CUMS model group (distilled water, 10 mL/kg), HLTP low-dose group (1, 10 mL/kg), HLTP medium-dose group (2, 10 mL/kg), HLTP high-dose group (4, 10 mL/kg), and Fluoxetine group (Fluoxetine, 1 mg/mL Flu, 10 mL/kg). Rats in the Normal group were group-housed (5 per cage) with unrestricted access to water and food. In contrast, rats in the remaining five groups were singly housed (1 per cage) and subjected to the CUMS protocol to induce depressive-like behavior, with unrestricted availability of food and water, excluding designated stress procedures.

### 2.4 Behavioral tests

Behavioral assessments were conducted from days 42 to 49 (n = 10 per group) in the following order: Sucrose Preference Test (SPT), Forced Swim Test (FST), Novelty-Suppressed Feeding Test (NSFT), and Open Field Test (OFT), the administration personnel were separated from the behavioral testers. Following the final drug administration on day 49, all rats were euthanized for subsequent analyses. Detailed experimental procedures are available in Supplementary Material.

### 2.5 Neuropathological examination

After fixation, brain tissues were sequentially dehydrated using graded ethanol and xylene, embedded in paraffin, and sectioned at a thickness of 4 μm. Neuronal morphology in the hippocampal CA1 region was examined using hematoxylin-eosin (HE) and Nissl staining under a light microscope (Olympus BX53).

### 2.6 Biochemical analyses

#### 2.6.1 HPLC quantification of NE, DA, and 5-HT

Three grams of brain tissue (The whole brain, similarly hereinafter.) were dissolved in 9 mL of physiological saline. A 4% perchloric acid aliquot was combined with 1 mL of the homogenate, vortexed for 30 s, and centrifuged at 9,600 *g* for 10 min at 4°C. A 0.22 μm membrane was used to filter the resultant supernatant before it was used for analysis. Using an Agilent Poroshell 120 EC-C18 column (4.6 × 150 mm, 4 μm) at 30°C, chromatographic separation was carried out. Delivered at a rate of 1.0 mL/min, the mobile phase is made up of 0.1% formic acid and methanol (93:7, v/v). The experiment was run for 15 min at 254 nm using a 20 μL injection volume.

#### 2.6.2 ELISA measurement of CRH, CORT, and ACTH

Following the instructions provided by the manufacturer, ELISA kits that are commercially available were used to measure the levels of CRH, CORT, and ACTH in both serum and brain tissues.

#### 2.6.3 Western Blot analysis of TNF-α, IL-1β, IL-10, BDNF, and CREB

Following the homogenization of 100 mg of brain tissue in 1 mL of ice-cold RIPA lysis buffer, the homogenates were subjected to centrifugation at 12,000 rpm for 15 min at 4°C. The protein concentrations in the resultant supernatants were quantified utilizing a BCA protein assay. The protein samples were transferred to PVDF membranes, blocked with 5% skim milk, and incubated overnight at 4°C with primary antibodies targeting TNF-α, IL-1β, IL-10, BDNF, CREB, and β-actin. After three washes with Tris-buffered saline containing 0.1% Tween-20 (TBST), the membranes were incubated for 2 hours at room temperature with HRP-conjugated goat anti-rabbit secondary antibodies. Following three supplementary washes with TBST, the protein bands were visualized employing an enhanced chemiluminescence detection system and quantified through grayscale densitometric analysis utilizing ImageJ software.

### 2.7 16S rRNA analysis of rat gut microbiota

The rat gut microbiota composition was examined using 16S rRNA high-throughput sequencing conducted by Wuhan Maiwei Biological Technology Co., Ltd. Samples of intestinal contents were obtained from the Control, CUMS, and CUMS-HLTP (high-dose HLTP) groups for sequencing. The CTAB method was used to extract genomic DNA, and its purity and concentration were determined before amplification. Polymerase Chain Reaction (PCR) amplification was conducted, followed by magnetic bead purification and enzymatic quantification. After successful library construction and quality assessment, sequencing was carried out. Raw sequence data were filtered, assembled, and aligned to generate high-quality valid reads. These reads were used for taxonomic annotation to determine microbial composition at various taxonomic levels. Microbial diversity and intergroup differences were evaluated using alpha and beta diversity indices, and differential taxa were identified using Linear Discriminant Analysis Effect Size (LEfSe) analysis.

### 2.8 Serum metabolomics analysis in rats

Untargeted metabolomics analysis was performed by Wuhan Metware Biotechnology Co., Ltd. (Wuhan, China). Serum samples from the Control, CUMS, and CUMS-HLTP (high-dose) groups underwent analysis via UPLC-Q-TOF-MS. Raw data were processed through format conversion, peak extraction, alignment, and normalization. Metabolite identification was achieved by integrating multiple public databases. Quality control (QC) samples were inserted after every 10 test samples to ensure data reliability. Subsequent data analyses were conducted using R software, including Principal Component Analysis (PCA), Orthogonal Partial Least Squares Discriminant Analysis (OPLS-DA), permutation tests, and loading analysis via S-plots. Potential biomarkers were identified based on accurate mass-to-charge ratios using the Human Metabolome Database (HMDB) and METLIN. Pathway enrichment analysis of differential metabolites was conducted using the MetaboAnalyst platform.

### 2.9 Correlation analysis among differential metabolites, differential microbiota, behavioral and biochemical indicators

Correlation analyses were identified among serum differential metabolites, screened differential microbiota, and behavioral/biochemical indicators using Pearson correlation coefficients. P-values were calculated, and the strength and direction of correlations were visualized as heatmaps.

### 2.10 Statistical analysis

All statistical analyses were carried out using SPSS software, version 19.0. The data was displayed as mean ± SD. Tukey’s HSD test was employed following one-way ANOVA to perform pairwise comparisons of intergroup differences. A P-value of 0.05 or less was considered statistically significant.

## 3 Results

### 3.1 HLTP alleviates depressive-like behaviors and protects hippocampal neurons in CUMS rats

**FIGURE 1:**
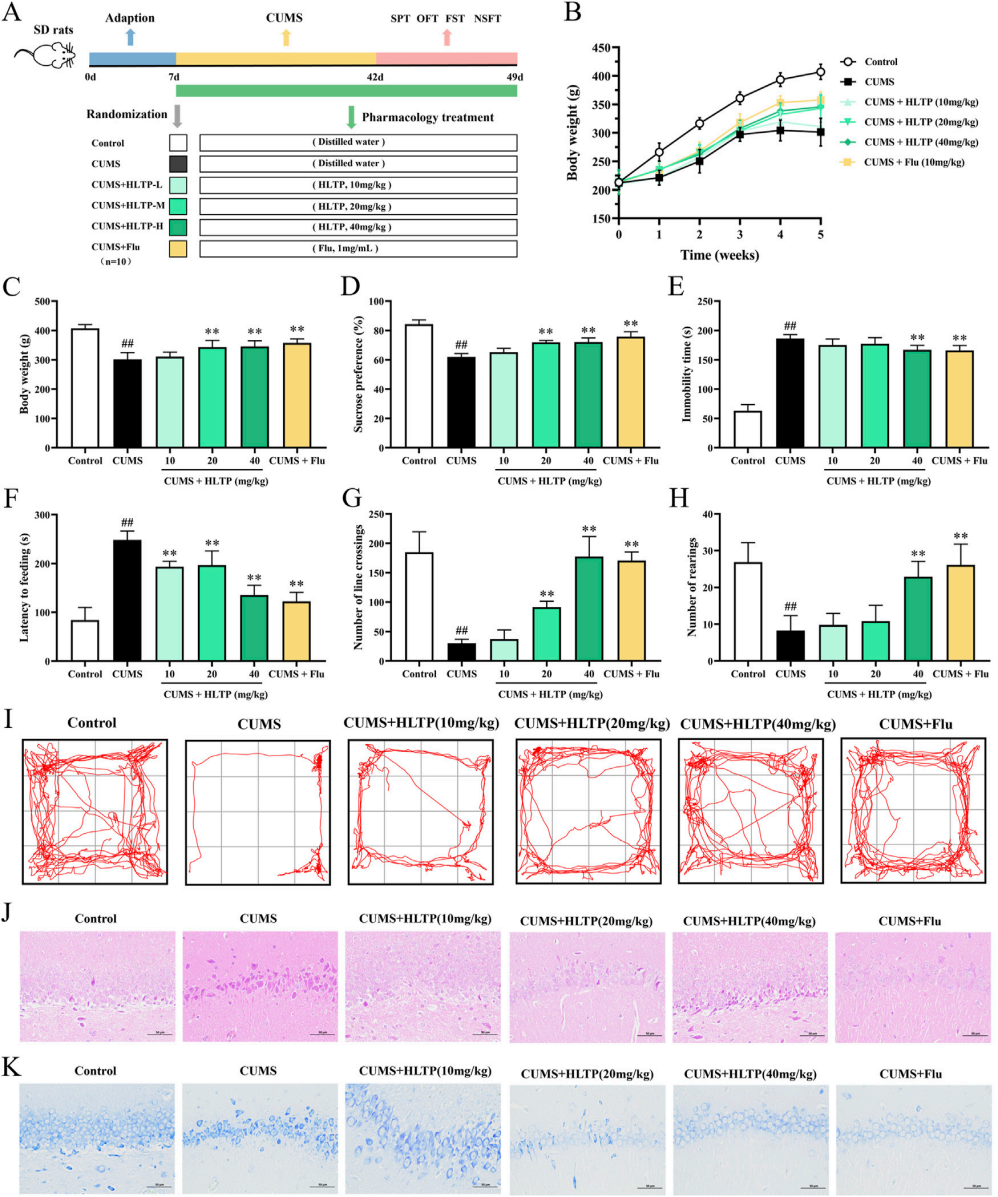
Effects of HLTP on depression behaviors and hippocampal neurons in CUMS rats. **(A)** Experimental flowchart. **(B)** Body weight changes. **(C)** Final average body weight. **(D)** Sucrose preference in SPT. **(E)** Immobility time in FST. **(F)** Latency to feed in Latency feeding test. **(G)** Number of crossings in OFT. **(H)** Number of rearings in OFT. **(I)** Movement trajectory in OFT. **(J, K)** HE and Nissl staining images. All data are expressed as the mean ± SD, n = 10 for each group, ^#^ *P* < 0.05, ^##^ *P* < 0.01 vs. Control; *P < 0.05, **P < 0.01 vs. CUMS.

The experimental design is illustrated in Figure 1A. Behavioral assessments were conducted following the drug intervention. After 5 weeks of CUMS exposure, rats in the HLTP-treated groups exhibited a significant increase in body weight compared with the model group (Figures 1B,C). Depression-like behaviors were evaluated using the SPT, FST, and OFT. The observational results indicated that rats exposed to CUMS displayed a decreased preference for sucrose solution, whereas HLTP treatment significantly improved SPT scores (Figure 1D). In the FST, subjects in the HLTP group demonstrated decreased immobility time relative to those in the CUMS group (Figure 1E). The CUMS group demonstrated a significant increase in ingestion latency, which was effectively reduced by HLTP treatment (Figure 1F). Furthermore, the CUMS group showed diminished locomotor activity, characterized by reductions in bottom square crossings and upright postures. HLTP treatment alleviated these motor deficits in a dose-dependent manner, with the 40 mg/kg HLTP and fluoxetine groups exhibiting comparable efficacy (Figures 1G–I). Collectively, these results suggest that HLTP enhances locomotor activity and significantly alleviates depression-like behaviors in CUMS-exposed rats.

Pyramidal neurons in the hippocampal CA1 region play a core hub role in the pathological process of depression, which is closely related to the morphological changes of the neurons themselves (Xie et al., 2024), neural circuit disorders (Ma et al., 2021), and abnormal molecular regulation (Cong et al., 2025). The pyramidal neurons in the CA1 region of patients with depression and model animals showed a significant reduction in dendritic complexity. The study of pyramidal neurons in the hippocampal CA1 region has opened up a new perspective for the clinical diagnosis and treatment of depression, linking microscopic cytopathology with macroscopic behavioral phenotypes, and providing a scientific basis for the development of targeted therapeutic strategies (Jia et al., 2024). This study adopted H&E staining and Nissl staining to observe the morphological changes of neurons, and the results are shown in Figures 1J,K. In comparison with the normal group, the CUMS group displayed disorganized and sparse pyramidal cell arrangements, with the majority of neuronal nuclei appearing shrunken. Notable atrophy, degeneration, and a reduction in both total and Nissl-positive cell counts were observed, along with poorly defined cell outlines. HLTP treatment partially reversed these pathological changes. Specifically, neuronal morphology in the HLTP-treated groups exhibited more normalized characteristics, with regularly shaped and densely arranged cells, and an increased number of Nissl bodies (The quantitative results are shown in Supplementary Figure S2). These findings indicate that HLTP attenuates hippocampal neuronal damage and apoptosis induced by CUMS in depressed rats.

### 3.2 HLTP ameliorates CUMS-induced dysregulation of neurotransmitters, HPA axis hormones, inflammatory cytokines, and neurotrophic factors in rats

**FIGURE 2:**
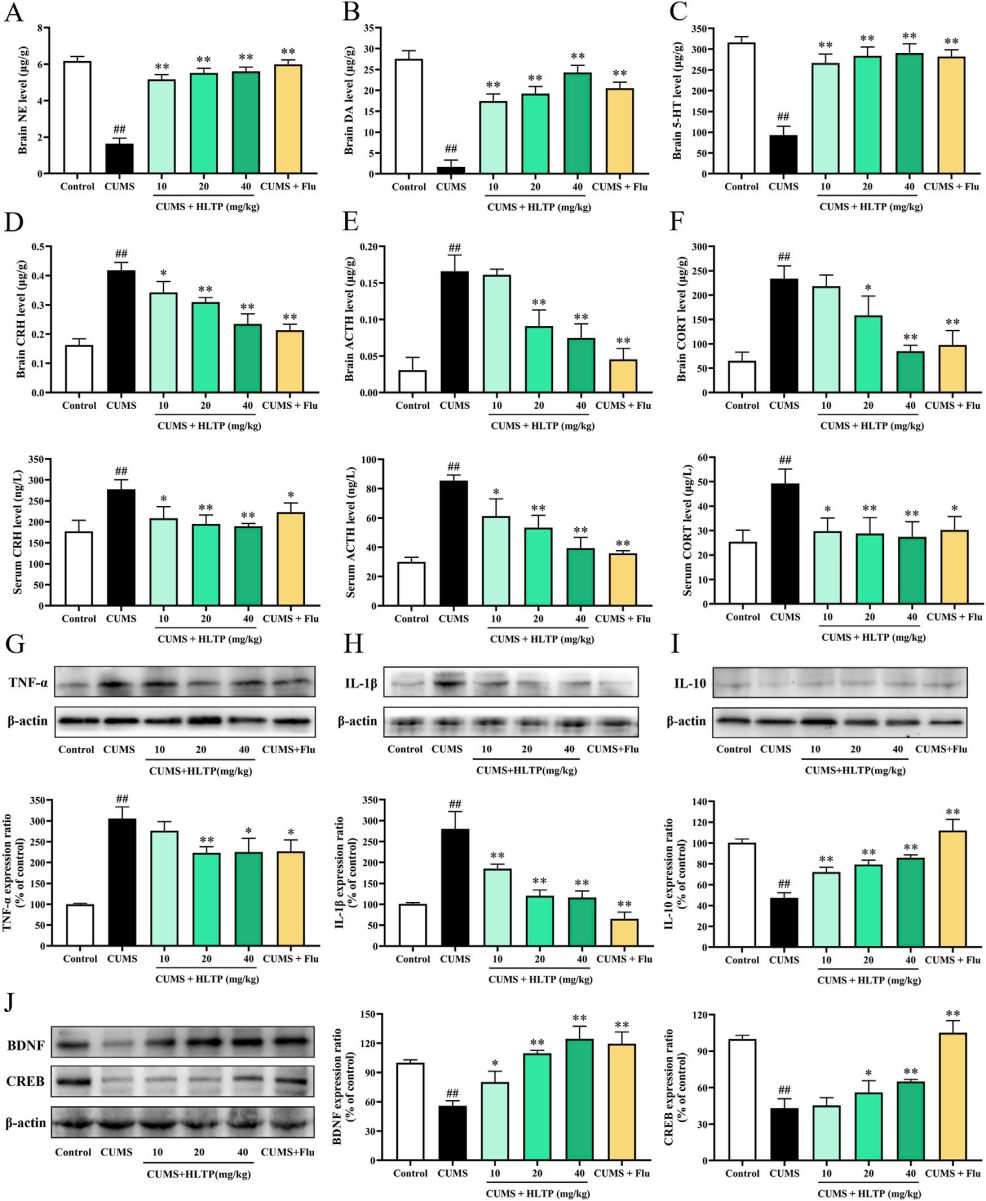
Effects of HLTP on Biochemical Parameters in CUMS Rats. The levels of monoamine neurotransmitters **(A)** NE, **(B)** DA, and **(C)** 5-HT in the brain tissues. The levels of **(D)** CRH, **(E)** ACTH, and **(F)** CORT in both serum and brain tissues. Treated HLTP, the expression of the brain protein levels of **(G)** TNF-α levels, **(H)** IL-1β, **(I)** IL-10, **(J)** BDNF and CRE. All data are expressed as the mean ± SD, n = 3 for each group, ^#^ *P* < 0.05, ^##^ *P* < 0.01 vs. Control; *P < 0.05, **P < 0.01 vs. CUMS.

Figures 2A–C shows how HLTP regulates the monoamine neurotransmitters DA, NE, and 5-HT in brain tissues. The levels of DA, NE, and 5-HT were significantly increased by the HLTP intervention in comparison to the CUMS group, indicating that HLTP has antidepressant-like effects by reestablishing the balance of monoamine neurotransmitters. Additionally, HLTP was found to modulate HPA axis dysfunction (Figures 2D–F). CUMS exposure resulted in markedly elevated levels of CRH, ACTH, and CORT in both serum and brain tissues relative to the Control group. In contrast, HLTP administration at doses of 10, 20, and 40 mg/kg significantly and dose-dependently reduced the levels of these hormones. HLTP decreased the levels of pro-inflammatory cytokines TNF-α and IL-1β, while markedly elevating the level of the anti-inflammatory cytokine IL-10 (Figures 2G–I). These observations reveal that HLTP mitigates CUMS-induced inflammatory damage by suppressing pro-inflammatory signaling and enhancing anti-inflammatory responses. Furthermore, HLTP upregulated the protein expression of BDNF and CREB within the brain tissues of rats subjected to CUMS-exposed, suggesting its potential to reverse neurotrophic impairments associated with depressive states (Figure 2J).

### 3.3 HLTP regulates gut microbial composition in CUMS rats

Gut microbiota is fundamentally involved in the onset of depression-like behaviors. In this study, intestinal contents from rats were evaluated via 16S rRNA genomic sequencing. Given that the 40 mg/kg HLTP dose exhibited the most significant effects on depression-like behaviors and biochemical markers—including monoamine neurotransmitter levels—in CUMS rats, we selected this dose to further explore its impact on gut microbial composition and serum metabolite profiles.

#### 3.3.1 Alpha and beta diversity analyses

**FIGURE 3:**
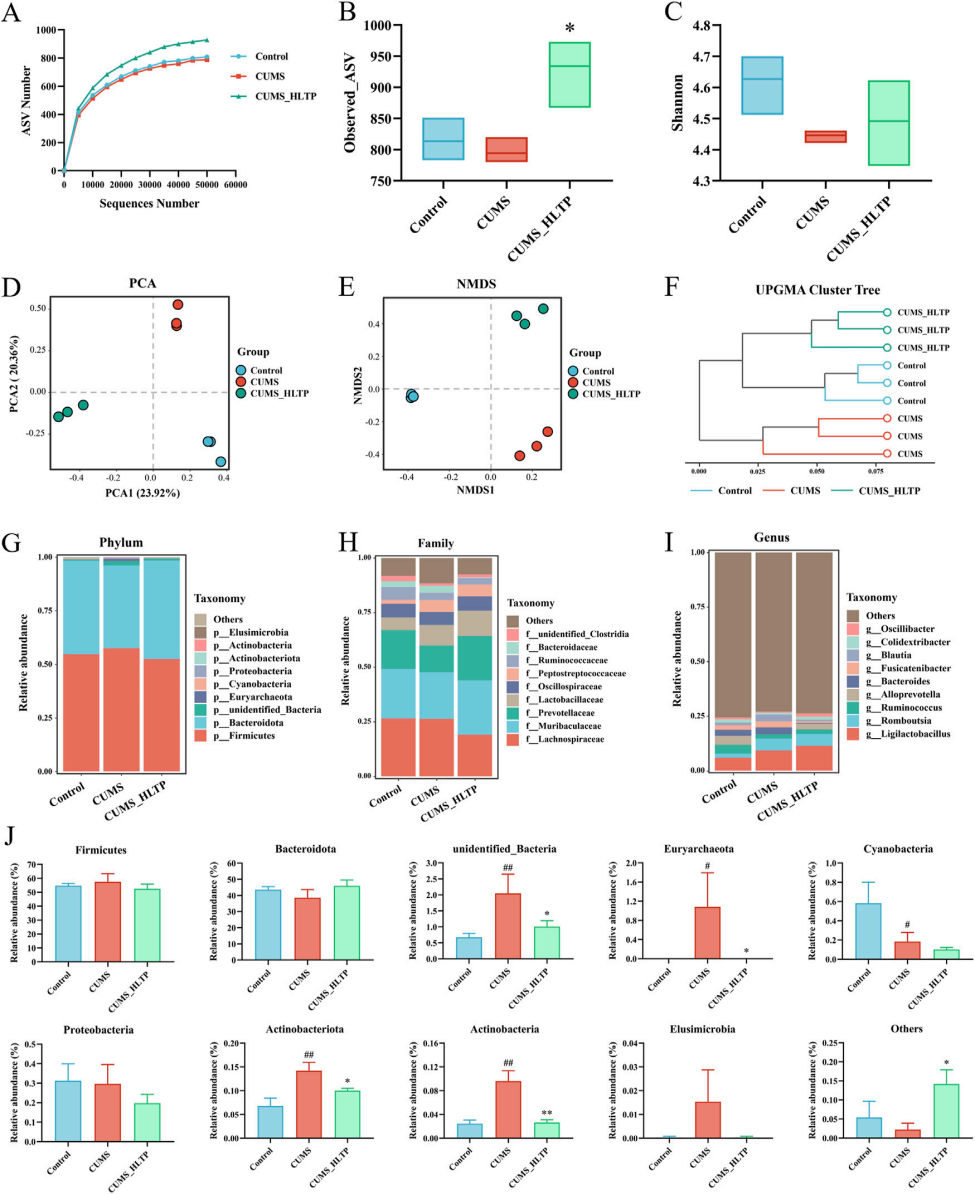
Effects of HLTP on gut microbiota in CUMS rats. **(A)** Rarefaction curve. **(B)** Observed_ASV index. **(C)** Shannon index. **(D)** PCA. **(E)** NMDS analysis. **(F)** UPGMA clustering tree analysis. **(G)** Relative abundance at the phylum level. **(H)** Relative abundance at the family level. **(I)** Relative abundance at the genus level. **(J)** Relative abundance of top 10 bacterial phyla in intestinal contents of each group. All data are expressed as the mean ± SD, n = 3 for each group, ^#^ *P* < 0.05, ^##^ *P* < 0.01 vs. Control; *P < 0.05, **P < 0.01 vs. CUMS.

Gut microbial alterations are closely linked to depression-like behaviors. In this experiment, 16S rRNA sequencing was applied to investigate shifts in the intestinal microbiota of rats (Yu et al., 2022). As 40 mg/kg HLTP demonstrated the most pronounced behavioral and biochemical effects in CUMS rats, we focused on analyzing the associated changes in gut flora and serum metabolites at this dosage. As shown in Figure 3A, rarefaction curves plateaued with increasing sequencing depth across all groups, indicating sufficient sequencing coverage for accurate microbiota profiling. Alpha diversity was analyzed via the Observed_ASV and Shannon indices at the ASV level (Figures 3B,C). Relative to the Control group, exposure to CUMS significantly reduced both species richness and microbial diversity. Conversely, treatment with HLTP notably restored both species richness and microbial diversity. To further assess microbial community structure, beta diversity analyses were conducted using PCA, NMDS, and UPGMA clustering (Figures 3D–F). PCA plots revealed an evident distinction between the Control and CUMS model groups, indicative of distinct microbial profiles. Notably, samples from the HLTP-treated group clustered more closely with those of the Control group in PCA2 dimensions, while remaining distinctly separated from the model group. Although the spatial distribution of the CUMS group in the NMDS2 dimension was closer to the blank Control group in the NMDS analysis, the HLTP-treated group showed an obvious trend of overall microbiota structure shift and shared more microbial characteristics with the Control group, indicating that HLTP intervention can effectively regulate the microbiota structure to restore it toward the normal state. UPGMA dendrograms corroborated these findings, showing that the Control and HLTP-treated groups formed a coherent cluster. Collectively, these findings suggest that HLTP effectively modulates gut microbial community structure disrupted by CUMS exposure.

#### 3.3.2 Species distribution analysis

To gain a better understanding of how HLTP affects gut microbial composition in CUMS-induced rats, we compared the relative abundances of microbial taxa at the phylum, family, and genus levels across experimental groups. Firmicutes and Bacteroidota dominated the phylum level, accounting for more than 90% of the total microbial population across all groups. The most abundant families were Lachnospiraceae, Muribaculaceae, and Prevotellaceae, which accounted for more than 60% of the total relative abundance. At the genus level, Ligilactobacillus remained consistently abundant, with its proportion exceeding 6% across all groups. Further examination of the top 10 most abundant phyla revealed notable alterations in microbial composition (Figure 3J). Specifically, in comparison with the Control group, the CUMS group showed marked increases in the proportional abundances of *unidentified_Bacteria*, Euryarchaeota, Actinobacteriota, and Actinobacteria. However, HLTP treatment attenuated the abundance of these four taxa relative to the CUMS group, while markedly elevating the cumulative relative abundance of other phyla. These findings indicate that HLTP treatment partially restores the CUMS-induced alterations in gut microbial composition at the phylum level in depressed rats.

#### 3.3.3 LEfSE analysis

**FIGURE 4:**
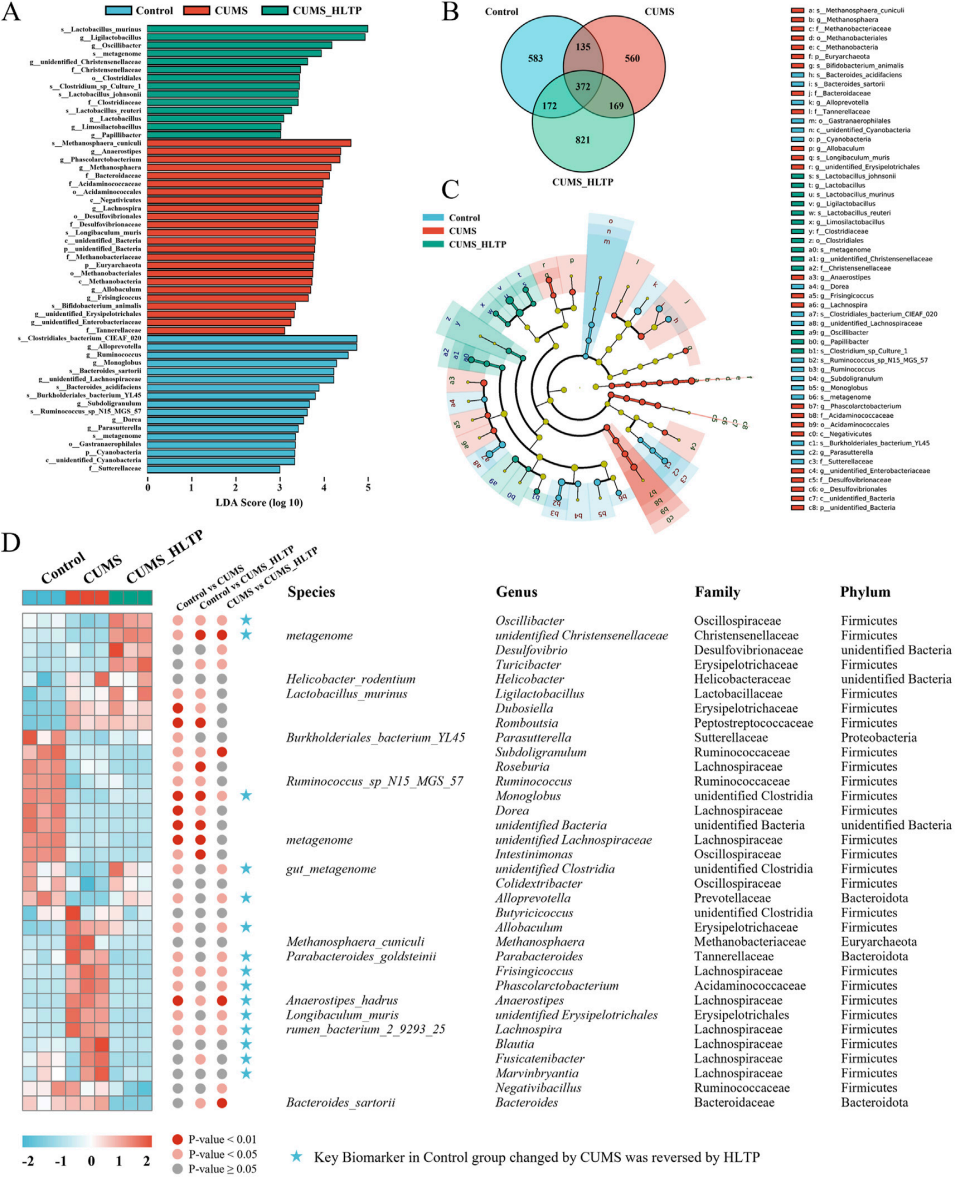
Analysis of differential microbiota. **(A)** Histogram of LDA scores based on ASVs; **(B)** Venn diagram based on ASVs; **(C)** Cladogram based on ASVs; **(D)** Heatmap showing relative abundance of microbiota reversed by HLTP intervention.

To identify microbial taxa exhibiting significant differences in abundance among groups, we first examined the shared and unique ASVs (Figure 4B). The Control, CUMS, and HLTP-treated groups contained 583, 560, and 821 unique ASVs, respectively. To further pinpoint group-specific microbial biomarkers with statistically significant differences, LEfSe was performed (LDA score >3; Figure 4A). This analysis identified 17, 24, and 14 significantly discriminant taxa in the Control, CUMS, and HLTP groups, respectively. These taxa were visualized using a cladogram, which illustrated the phylogenetic relationships among differentially abundant microbial lineages (Figure 4C). Additionally, heatmap analysis (Figure 4D) demonstrated that HLTP treatment significantly reversed the abundance of 15 out of 34 differential bacterial taxa when compared to the CUMS group. Notably, HLTP administration led to reductions in the abundance of *Marvinbryantia*, *Fusicatenibacter*, *Blautia*, *Phascolarctobacterium*, *Frisingicoccus*, *Lachnospira*, *Anaerostipes*, *Parabacteroides*, *Allobaculum*, and *unidentified_Erysipelotrichales.* In contrast, it increased the relative abundance of *Oscillibacter*, *Alloprevotella*, *Monoglobus*, *unidentified_Clostridia*, and *unidentified_Christensenellaceae.* These results indicate that HLTP modulates the gut microbiota composition disrupted by CUMS exposure, shifting it towards a profile exhibiting greater similarity to that of the healthy control group.

### 3.4 Effects of HLTP on serum metabolic profile in CUMS-induced depressed rats

**FIGURE 5:**
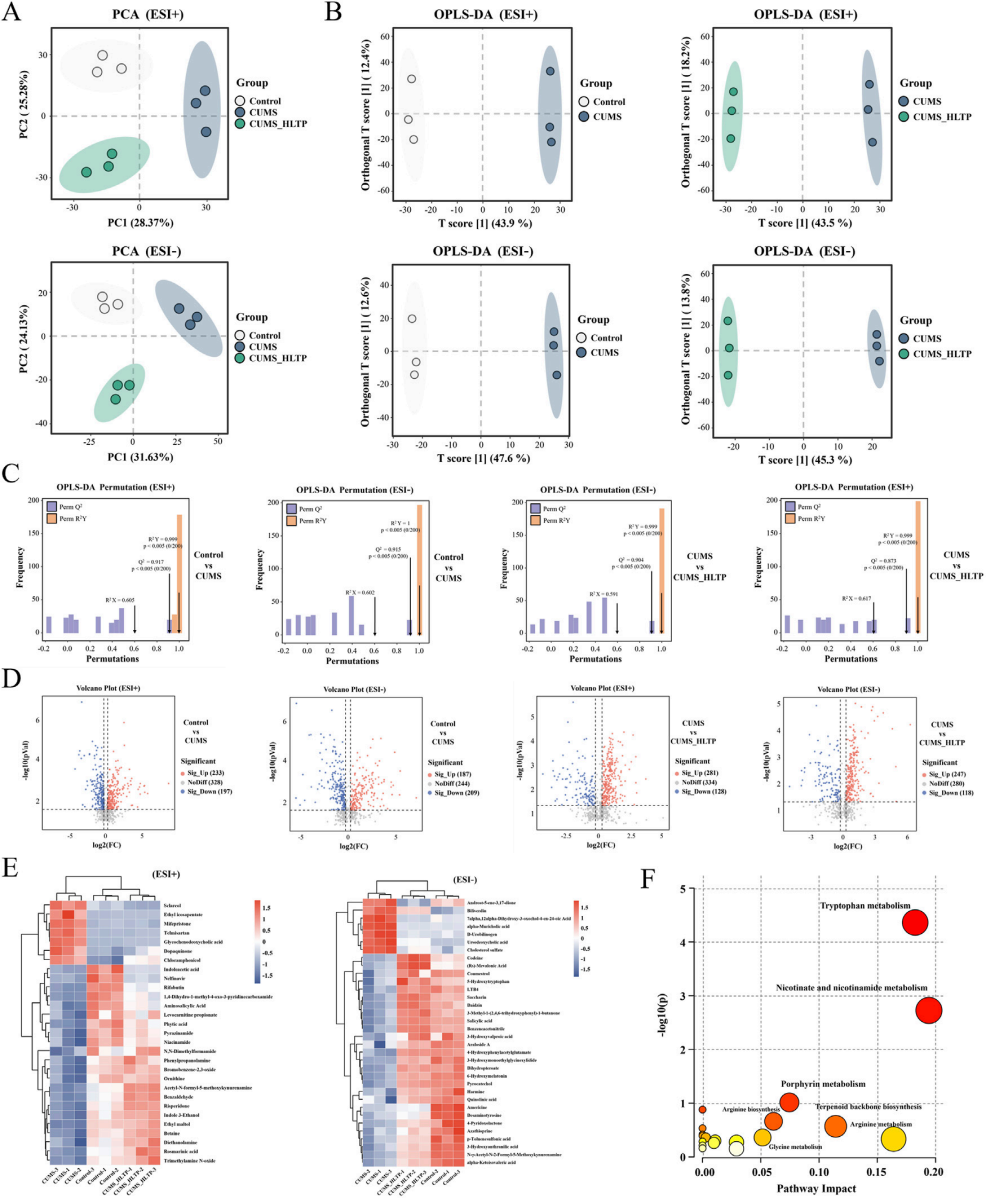
Effects of HLTP on serum metabolites in CUMS rats. In positive and negative ion modes **(A)** PCA. **(B)** OPLS-D. **(C)** OPLS-DA. **(D)** Volcano plots (red: significantly upregulated metabolites. blue: significantly downregulated metabolites). **(E)** Heatmap showing relative abundance changes of differential metabolites. **(F)** Metabolic pathways associated with differential metabolites. n = 3 for each group.

Serum metabolites were analyzed using UPLC-Q-TOF-MS to ascertain whether HLTP alters the metabolic profile in rats with CUMS-induced depression. Significant differences in the three groups' overall metabolic profiles were revealed by PCA, which showed clear separation between them in both positive and negative ionization modes (Figure 5A).

Supervised orthogonal partial least squares-discriminant analysis (OPLS-DA) was implemented to further characterize metabolic changes. The resulting score plots demonstrated clear intergroup distinctions across both ion modes, with T score1 values of 43.9%, 47.6%, 43.5%, and 45.3%, respectively (Figure 5B). Model validation via 200 permutation tests confirmed high reliability, with R^2^X > 0.59, R^2^Y > 0.99, and Q^2^ > 0.87 across all four models, supporting the reliability and predictive power of the OPLS-DA analyses (Figure 5C).

Differential metabolites were screened using volcano plots according to the specified parameters of Variable Projection Importance (VIP) > 1.0, *P* < 0.05, and fold change (FC) >1.5 or <0.8 (Figure 5D). Metabolites were further refined by intersecting those significantly altered in the two comparison groups under both ionization modes and identifying those in the HLTP group that shifted toward levels observed in the healthy control group. As a result, under the positive and negative ion modes, respectively, 29 and 34 different metabolites were found. These metabolites were subsequently annotated using the KEGG and HMDB (Supplementary Table S1). A heatmap was constructed to illustrate the relative abundance of these metabolites, with blue-to-red gradients reflecting increasing metabolite levels. Notably, HLTP treatment significantly normalized the levels of 29 metabolites in positive ion mode (e.g., indoleacetic acid) and 34 metabolites in negative ion mode (e.g., 5-hydroxytryptophan) (Figure 5E). To elucidate the potential biochemical pathways affected by HLTP, KEGG pathway enrichment analysis was conducted. Among the identified metabolites, 22 were enriched in 19 metabolic pathways, with 10 pathways exhibiting impact values greater than zero (Figure 5F; Supplementary Table S2). These pathways include tryptophan metabolism, nicotinate and nicotinamide metabolism, porphyrin metabolism, arginine biosynthesis, terpenoid backbone biosynthesis, glycine metabolism, arginine metabolism, valine degradation, arachidonic acid metabolism, and Primary bile acid biosynthesis. Collectively, these results indicate that HLTP may confer its antidepressant effects through the regulation of multiple metabolic pathways altered in CUMS-induced depression.

### 3.5 Correlation analysis of differential metabolites, gut microbiota, and behavioral and biochemical indices

**FIGURE 6:**
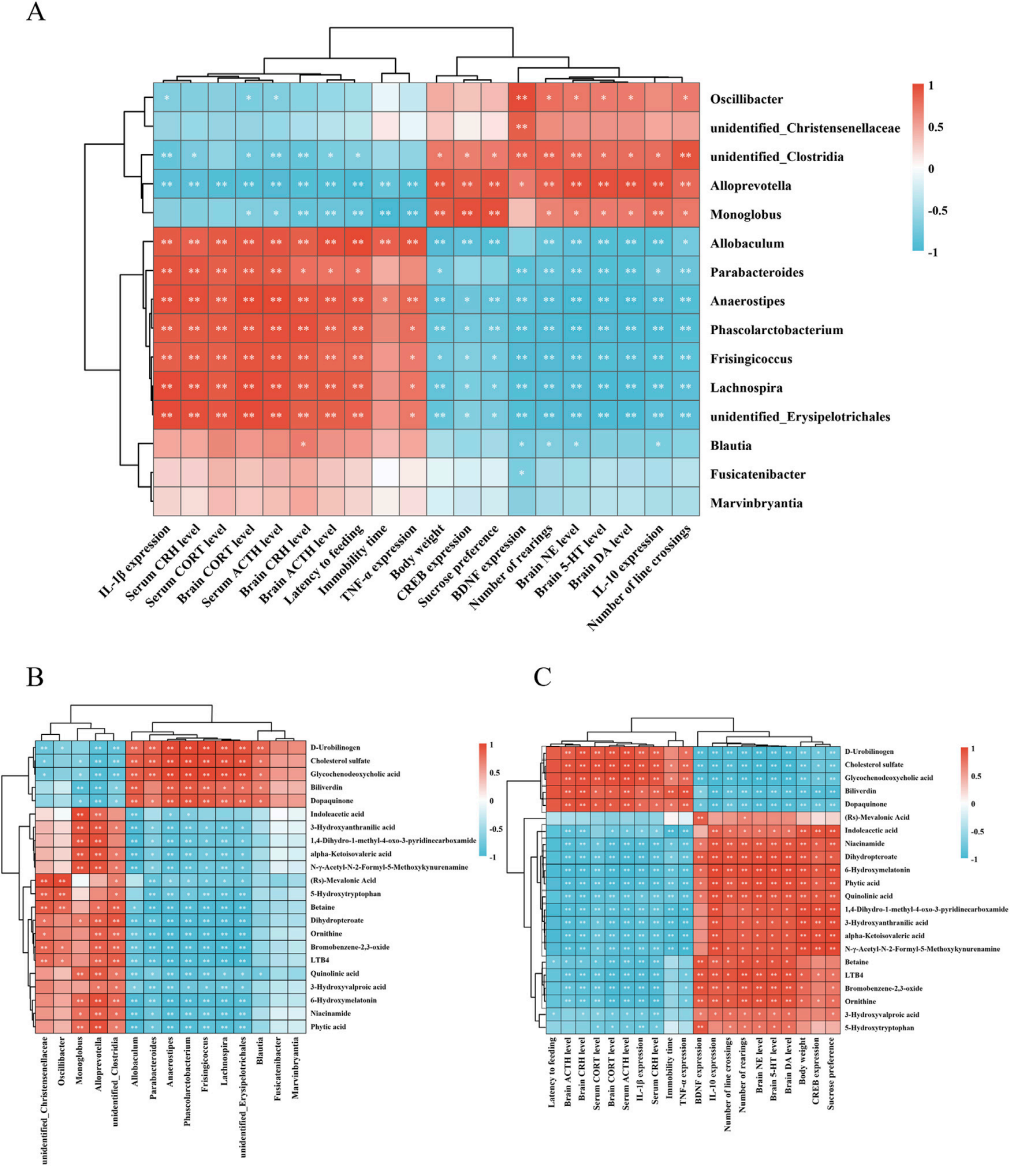
Correlation analysis of differential metabolites with gut microbiota, behavioral and biochemical indices. Heatmap of correlation analysis between **(A)** differential gut microbiota, behavioral and biochemical indices. **(B)** differential metabolites and gut microbiota. **(C)** differential metabolites, behavioral and biochemical indices. *P < 0.05, **P < 0.01. n = 3 for each group.

To investigate the relationships between 15 distinct gut microbial genera and 20 behavioral and biochemical parameters, a Pearson correlation analysis was performed (Figure 6A). Significant positive correlations (*P* < 0.05 or *P* < 0.01) were observed between levels of monoamine neurotransmitters (5-HT, DA, NE), the anti-inflammatory cytokine IL-10, neurotrophic factors (BDNF and CERB), body weight, sucrose preference rate, open-field crossings, and rearing times with the genera *Oscillibacter*, *unidentified_Christensenellaceae*, *Monoglobus*, *unidentified_Clostridia*, and *Alloprevotella*. In contrast, these behavioral and biochemical indicators were negatively correlated with *Anaerostipes*, *unidentified_Erysipelotrichales*, *Lachnospira*, *Blautia*, and *Fusicatenibacter*. Conversely, HPA axis-related hormones (CRH, ACTH, CORT), pro-inflammatory cytokines (TNF-α, IL-1β), feeding latency, and immobility time in the forced swimming test exhibited positive correlations with *Frisingicoccus*, *Phascolarctobacterium*, *Blautia*, *Fusicatenibacter*, and *Marvinbryantia*, but were negatively associated with *Monoglobus*, *unidentified_Clostridia*, and *Alloprevotella*.

Pearson correlation analysis was further conducted between 22 differential metabolites and gut microbiota (Figure 6B). The results revealed that metabolites upregulated by HLTP were positively correlated with enriched microbial taxa, such as *Alloprevotella* and *unidentified_Clostridia*. For example, phytic acid, nicotinamide, and 6-hydroxymelatonin exhibited significant positive correlations with these taxa, while showing negative correlations with microbial genera downregulated by HLTP, such as *Parabacteroides* and *Anaerostipes*. Conversely, metabolites that were downregulated following HLTP treatment exhibited opposite correlation trends.

Correlation analysis between differential metabolites and behavioral or biochemical parameters (Figure 6C) demonstrated that HLTP-upregulated metabolites were positively associated with improved neurochemical indices, including elevated levels of 5-HT, DA, and NE in brain tissue, and negatively associated with pathological parameters such as immobility time in the forced swimming test and serum levels of CRH and ACTH. In contrast, HLTP-downregulated metabolites were positively correlated with adverse behavioral and biochemical parameters, and negatively correlated with the a forementioned beneficial indices.

Collectively, these results imply that HLTP may mediate its antidepressant effects through the modulation of gut microbiota to regulate host metabolic pathways, thereby alleviating behavioral disturbances and restoring normal neurochemical and endocrine function in the brain and peripheral systems.

## 4 Discussion

Depression ranks among the most widespread psychiatric disorders worldwide and is defined by core symptoms such as persistent low mood, anhedonia, and reduced volitional activity (Qiao et al., 2020). The CUMS paradigm reliably recapitulates both behavioral phenotypes and key biochemical features of human depression and is therefore widely employed in preclinical depression research (Sharma and Thakur, 2015). In the current study, rats that underwent CUMS exhibited hallmark depressive-like behaviors, including reduced body weight, decreased sucrose preference in the SPT, prolonged immobility in the FST, increased latency to feed in the NSFT, and diminished locomotor activity in the OFT. Notably, HLTP treatment significantly ameliorated these behavioral abnormalities, as reflected by improved digestive and absorptive capacity (Jung et al., 2017), restoration of sucrose preference (Gross and Pinhasov, 2016), and enhancement of general activity levels (Brenes Sáenz et al., 2006), thereby demonstrating robust antidepressant-like effects.

The monoaminergic system serves as a pivotal component in the pathophysiology of depression. Specifically, serotonin (5-HT) is critical for emotional regulation (Goda et al., 2015); NE contributes to learning, memory, and emotional reactivity (Root et al., 2015); and DA is essential for cognitive processing and motivation (Rominger et al., 2015). Furthermore, hyperactivity of the HPA axis is a well-established neuroendocrine hallmark of depression, with elevated levels of CRH, ACTH, and CORT commonly observed in the cerebrospinal fluid of depressed patients (Nemeroff et al., 1984). This work detected that HLTP intervention significantly increased the levels of DA, NE and 5-HT in rat brain tissues, and decreased the levels of CRH, ACTH and CORT in serum and brain tissues, which objectively reflected the changing trends of monoamine neurotransmitters and HPA axis-related hormones. It should be noted that the current study is at the level of index detection, and the specific molecular mechanisms have not been deeply explored through direct experiments (such as key enzyme activity determination, receptor function verification, signaling pathway protein detection, *etc.*). The relevant mechanisms will be further investigated in subsequent experiments. Additionally, it is worth noting that the academic community currently believes that the blood-brain barrier has a low permeability to 5-HT, making it difficult for peripheral 5-HT to directly enter the brain in large quantities. In this study, although the HLTP-induced gut microbiota is a potential source of peripheral 5-HT production, we speculate that HLTP exerts its antidepressant effect not by relying on the direct passage of peripheral 5-HT through the blood-brain barrier into the brain. Combined with the results of this study, HLTP may affect the level of 5-HT in the brain through a variety of indirect mechanisms. (1) HLTP can regulate the MGBA and improve the structure of the gut microbiota, and changes in the gut microbiota can affect the synthesis and release of 5-HT by intestinal endocrine cells. These gut-derived 5-HT can regulate the function of the central nervous system through pathways such as the vagus nerve, indirectly affecting the metabolism and signal transduction of neurotransmitters in the brain (Layunta et al., 2021; Dicks, 2022). (2) HLTP may regulate the function of the HPA axis, reduce the levels of CRH, ACTH, and CORT in serum and brain tissue, reduce stress-induced damage to nerve cells, and provide a favorable microenvironment for the synthesis of monoamine neurotransmitters such as 5-HT in the brain (Higuchi et al., 2017). Finally, we speculate that HLTP is likely involved in the regulation of the synthesis or degradation of monoamine neurotransmitters in the brain. From the perspective of synthesis, HLTP may promote the synthesis of monoamine neurotransmitters by regulating the activity of related synthetic enzymes (such as tryptophan hydroxylase, which is involved in the synthesis of 5-HT); from the perspective of degradation, HLTP may be able to inhibit the activity of degradative enzymes such as monoamine oxidase, reducing the decomposition of monoamine neurotransmitters. In addition, HLTP also increases the levels of neurotrophic factors such as BDNF and CREB, which play important roles in neural plasticity and neurotransmitter metabolism regulation, and may indirectly participate in the regulation of the synthesis and degradation of monoamine neurotransmitters in the brain.

Emerging evidence increasingly characterizes depression as an inflammatory disorder driven by neuroimmune dysregulation, marked by elevated levels of pro-inflammatory cytokines and insufficient anti-inflammatory responses (Vollmer et al., 2015). This study detected that HLTP intervention could reduce the protein expression levels of TNF-α and IL-1β, and increase the protein expression level of IL-10, reflecting its impact on neuroinflammatory indices. It should be noted that the current data show the correlation between HLTP intervention and the improvement of neuroinflammation, and the specific molecular mechanisms and signaling pathways by which it inhibits neuroinflammation will be further explored in subsequent studies.

The neurotrophic hypothesis of depression implicates BDNF deficiency as a key contributor to the disorder’s pathogenesis (Martinowich and Lu, 2008), particularly in relation to region-specific structural and functional brain changes (Duman et al., 2016). Since the activation of cAMP response element-binding protein (CREB) is known to promote BDNF expression—and both CREB and BDNF levels are typically diminished in individuals with depression—our findings that HLTP significantly upregulated both BDNF and CREB suggest a potential antidepressant mechanism involving the restoration of neuroplasticity (Chen et al., 2001).

An increasing volume of evidence substantiates the hypothesis that modulation of gut microbiota represents a principal mechanism underlying the therapeutic effects of antidepressant agents (Kelly et al., 2019). For instance, administration of *Lactobacillus* probiotics has been demonstrated to ameliorate depressive-like behaviors in mice, indicating that structural regulation of gut microbial communities can ameliorate depressive symptoms (Marin et al., 2017). In our study, HLTP treatment led to marked alterations in gut microbial composition compared to the CUMS group. Specifically, HLTP significantly increased both microbial diversity and richness, while also modulating the relative abundance of multiple taxa across different phylogenetic levels. Compared with CUMS rats, HLTP reversed the abundance of 15 bacterial genera, including *Alloprevotella*, *Monoglobus*, and *Marvinbryantia*. Notably, the abundance of *Alloprevotella*—previously reported to correlate positively with serum 5-HT and BDNF levels (Meilan et al., 2021)—was significantly upregulated following HLTP treatment. Likewise, the reduction in *Monoglobus* abundance observed in CUMS-exposed rats was restored by HLTP, consistent with earlier findings (Liang et al., 2022). In contrast, *Marvinbryantia*, has been negatively associated with 5-HA and DA levels and observed at increased level in individuals with depression (Yu et al., 2017). A systematic analysis of gut microbial alterations in depression further revealed that at least three clinical studies reported an increased abundance of *Fusicatenibacter*, *Blautia*, *Parabacteroides*, *Lachnospira*, and *Anaerostipes* in depressed patients (Gao et al., 2023). In agreement with these observations, our data showed that HLTP significantly attenuated the CUMS-induced enrichment of these five genera. Additionally, HLTP regulated the abundance of *Frisingicoccus* (Zheng et al., 2022), *Allobaculum* (Watanabe et al., 2023), *Oscillibacter*, *unidentified_Clostridia*, *unidentified_Erysipelotrichales* (Barandouzi et al., 2020), *Phascolarctobacterium* (Choi et al., 2018), and *unidentified_Christensenellacea* (Hill-Burns et al., 2017), with directional changes aligned with observations reported in the literature. These results imply that in CUMS-induced depressed rats, HLTP may have antidepressant effects, at least in part, by altering the structural make up and diversity of the gut microbiota. Untargeted metabolomics analysis revealed that HLTP modulates the serum metabolic profile of CUMS rats by regulating 10 key metabolic pathways, including tryptophan metabolism, nicotinate/nicotinamide metabolism, and porphyrin metabolism. Among these, tryptophan metabolism plays a pivotal role in the pathophysiology of depression, as tryptophan deficiency has been strongly associated with the manifestation of depressive symptoms (Miura et al., 2008). In the present study, CUMS rats exhibited significantly reduced serum levels of five tryptophan-derived metabolites: 5-HTP (Liu et al., 2024), 3-hydroxyanthranilic acid, 6-hydroxymelatonin, N-γ-acetyl-N-2-formyl-5-methylkynurenine, and indoleacetic acid. As a direct precursor for serotonin, 5-HTP depletion may contribute to reduced serotonergic neurotransmission, thereby exacerbating depressive symptoms (Duan et al., 2006). Meanwhile, 6-hydroxymelatonin is known to exert neuroprotective effects by maintaining mitochondrial integrity and enhancing cellular viability. Notably, HLTP treatment effectively restored the levels of these metabolites, indicating a normalization of tryptophan metabolism. Furthermore, several tryptophan metabolites are intermediates in nicotinate and nicotinamide metabolism, a pathway that regulates cellular energy homeostasis, redox balance, and inflammatory responses (Wang et al., 2023). As important vitamin B3 derivatives, nicotinic acid and nicotinamide play critical roles in energy metabolism, DNA repair, cellular signal transduction, and other processes. In patients with depression, abnormalities in the levels of nicotinamide adenine dinucleotide (NAD+) and its phosphorylated form (NADP+) are commonly observed, and the synthesis of NAD+ and NADP+ is closely associated with the metabolism of nicotinic acid and nicotinamide (Liu et al., 2009). NAD+ is involved in redox reactions within the mitochondrial respiratory chain, providing energy for nerve cells. A decrease in its levels can lead to energy metabolic disorders in nerve cells, affecting the synthesis and release of neurotransmitters, which is associated with the occurrence and development of depression (Covarrubias et al., 2021). The porphyrin metabolic pathway is primarily involved in heme synthesis. Heme serves not only as a cofactor for important proteins such as hemoglobin and cytochromes but also participates in regulating intracellular redox status, signal transduction, and gene expression (Voltarelli et al., 2023). Studies have shown that porphyrin metabolism is abnormal in patients with depression. Impaired heme synthesis may increase oxidative stress levels in nerve cells, damage mitochondrial function, and further affect neurotransmitter metabolism and neural plasticity (Atamna and Frey, 2004). HLTP significantly modulated the levels of nicotinamide, 1,4-dihydro-1-methyl-4-oxo-3-pyridinecarboxamide, and quinolinic acid, suggesting that nicotinamide metabolism may constitute an additional pathway underlying HLTP antidepressant effects. Moreover, CUMS rats displayed elevated serum levels of D-urobilinogen and dehydrobilirubin, both of which have been associated with mitochondrial dysfunction, oxidative stress, and the induction of apoptosis (Doi and Horie, 2010).

Correlation analysis revealed strong interrelationships among HLTP-modulated serum metabolites, gut microbiota, behavioral outcomes, and biochemical indices. The gut microbiota has been demonstrated to regulate HPA axis activity, which in turn influences microbial composition and intestinal permeability (Viladomiu et al., 2013). In our study, bacteria downregulated by HLTP showed a positive association with elevated levels of HPA axis-related hormones (CRH, ACTH), indicating their involvement in neuroendocrine dysregulation. Certain microbial taxa are known to secrete lipopolysaccharides and peptidoglycans that activate systemic inflammatory responses, leading to the release of pro-inflammatory cytokines into circulation. These cytokines can cross the blood-brain barrier and contribute to the onset and progression of depressive symptoms (Stevens et al., 2018). Conversely, some beneficial gut microbes function as “neurotransmitter enhancers” by producing 5-HT, DA, and NA (Yunes et al., 2020), simultaneously promoting BDNF expression and neurogenesis (Satomura et al., 2011). For example, *Alloprevotella* exhibited significant positive correlations with monoamine neurotransmitters and BDNF, alongside negative correlations with pro-inflammatory cytokines, suggesting its prospective involvement in regulating central nervous system function via endocrine, immune, and metabolic mechanisms. The central nervous system can also influence gut microbiota composition directly via neural signaling pathways and HPA axis activation, or indirectly through systemic signaling molecules, thereby establishing a bidirectional MGBA (Lach et al., 2018). Notably, key HLTP-regulated tryptophan metabolites were strongly positively correlated with *Alloprevotella*, 5-HT, and BDNF levels, underscoring the integrated contributions of microbial homeostasis, neurotransmitter biosynthesis, and neurotrophic signaling to HLTP’s antidepressant effects.

**FIGURE 7:**
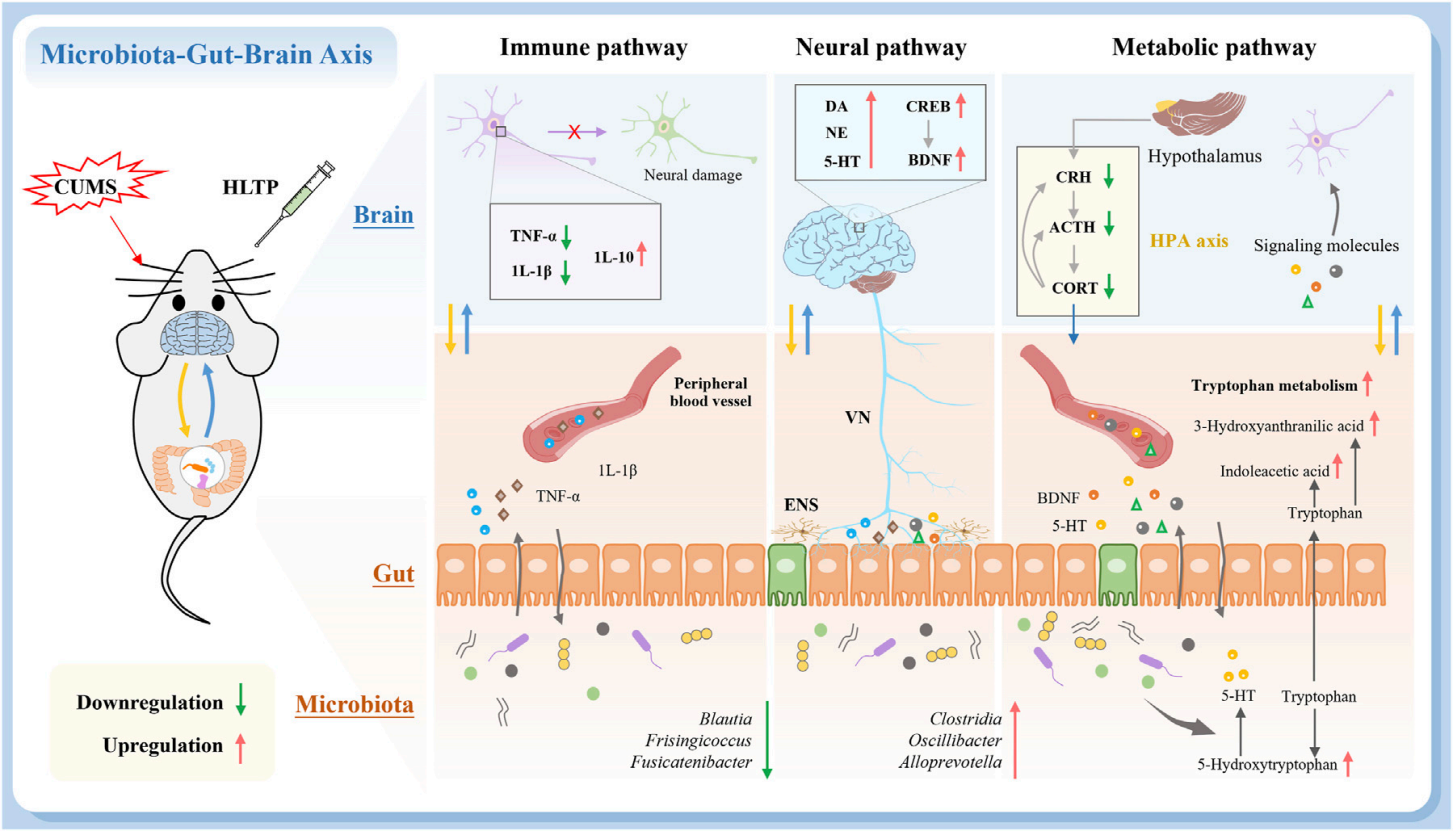
Schematic diagram of the antidepressant mechanism of HLTP. (Red and green arrows indicate upregulation and downregulation, respectively).

In summary, our results demonstrate that HLTP effectively alleviates depression-like behaviors in CUMS-induced rats by modulating gut microbiota to regulate key metabolic pathways, particularly tryptophan metabolism and nicotinate/nicotinamide metabolism. This microbial-metabolic intervention restores dysregulated serum metabolites and subsequently normalizes inflammatory cytokines, neurotrophic factors, endocrine hormones, and monoamine neurotransmitters. Collectively, these observations indicate that the antidepressant effects of HLTP are exerted via modulation of the MGBA (Figure 7). However, for the development and utilization of HLTP, further safety and efficacy experiments (such as acute and chronic toxicity tests, reproductive toxicity tests, *etc.*) are still required for verification.

## 5 Conclusion

In summary, this study employed a CUMS-induced depression model in rats to evaluate the antidepressant effects of HLTP and elucidate its underlying mechanisms. The results demonstrated that HLTP administration at varying doses: Ameliorated depression-like behaviors and prevented weight loss in CUMS rats. Attenuated neuronal damage in the hippocampal CA1 region. Modulated key biochemical markers, including Pro- and anti-inflammatory cytokines, Neurotrophic factors, Endocrine hormones, and Monoamine neurotransmitter levels. Furthermore, HLTP was shown to: Restore gut microbiota homeostasis by regulating: Microbial diversity, Taxonomic abundance, and Community structure. Reverse CUMS-induced metabolic disturbances, with strong correlations observed among: Differential serum metabolites, Altered gut microbial taxa, and Behavioral and biochemical indices. Collectively, these findings indicate that HLTP mediates its antidepressant effects via the modulation of the MGBA. The overall research results and conclusions of this paper are presented in the graphical abstract.

However, this study still has several limitations: Targeted analysis of serum prototype components and metabolites post-administration is required to identify the material basis of HLTP’s antidepressant efficacy. A placebo group should be incorporated, and functional magnetic resonance imaging (fMRI) employed to monitor brain regional activities, thereby further differentiating true therapeutic effects from placebo effects.

## Data Availability

The original contributions presented in the study are publicly available. This data can be found here: China National Center for Bioinformation (NGDC), “HLTP”. OMIX ID: OMIX011632. National Center for Biotechnology Information (NCBI), (2025). “Hemerocallis citrina Baroni leaf total phenol alleviates depressive-like behaviors via modulating “microbiota-gut-brain” axis in chronic unpredictable mild stress -induced rats”. Sequence Read Archive (SRA). BioProject: PRJNA1305707 (https://www.ncbi.nlm.nih.gov/bioproject/PRJNA1305707).

## Abbreviations

HLTP, Hemerocallis citrina Baroni leaf total phenol; CUMS, Chronic unpredictable mild stress; MGBA, Microbiota-gut-brain axis; HPA, Hypothalamic-Pituitary-Adrenal; TCM, Traditional Chinese medicine; 5-HT, 5-hydroxytryptamine; DA, Dopamine; NE, Noradrenaline; ELISA, Enzyme-linked immunosorbent assay; CREB, cAMP Response Element Binding Protein; CRH, Corticotropin-Releasing Hormone; ACTH, Adrenocorticotropic hormone; CORT, Corticosterone; BDNF, Brain-derived neurotrophic factor; TNF-α, Tumor necrosis factor-α; IL-1β, Interleukin-1β; IL-10, Interleukin-10; PCA, Principal Component Analysis; OPLS-DA, Orthogonal Partial Least Squares Discriminant Analysis; HE, Hematoxylin-eosin; SPT, Sucrose Preference Test; FST, Forced Swim Test; NSFT, Novelty-Suppressed Feeding Test; OFT, Open Field Test; UPLC-Q-TOF-MS/MS, Ultra-performance liquid chromatography coupled with quadrupole time-of-flight tandem mass spectrometry.

## References

[B1] Agirman G. Hsiao E. Y. (2021). SnapShot: the microbiota-gut-brain axis. Cell 184, 2524–2524.e1. 10.1016/j.cell.2021.03.022 33930299

[B2] Atamna H. Frey W. H. (2004). A role for heme in Alzheimer’s disease: heme binds amyloid beta and has altered metabolism. Proc. Natl. Acad. Sci. U. S. A. 101, 11153–11158. 10.1073/pnas.0404349101 15263070 PMC503755

[B3] Barandouzi Z. A. Starkweather A. R. Henderson W. A. Gyamfi A. Cong X. S. (2020). Altered composition of Gut microbiota in depression: a systematic review. Front. Psychiatry 11, 541. 10.3389/fpsyt.2020.00541 32587537 PMC7299157

[B4] Brenes Sáenz J. C. Villagra O. R. Fornaguera Trías J. (2006). Factor analysis of forced swimming test, sucrose preference test and open field test on enriched, social and isolated reared rats. Behav. Brain Res. 169, 57–65. 10.1016/j.bbr.2005.12.001 16414129

[B5] Chen B. Dowlatshahi D. MacQueen G. M. Wang J. F. Young L. T. (2001). Increased hippocampal BDNF immunoreactivity in subjects treated with antidepressant medication. Biol. Psychiatry 50, 260–265. 10.1016/s0006-3223(01)01083-6 11522260

[B6] Choi J. Lee S. Won J. Jin Y. Hong Y. Hur T. Y. (2018). Pathophysiological and neurobehavioral characteristics of a propionic acid-mediated autism-like rat model. PLoS One 13, e0192925. 10.1371/journal.pone.0192925 29447237 PMC5814017

[B7] Cichewicz R. H. Zhang Y. Seeram N. P. Nair M. G. (2004). Inhibition of human tumor cell proliferation by novel anthraquinones from daylilies. Life Sci. 74, 1791–1799. 10.1016/j.lfs.2003.08.034 14741736

[B8] Cong L. Ding S. Guo Y. Tian J. Liu L. Su M. (2025). Acupuncture alleviates CSDS-induced depressive-like behaviors by modulating synaptic plasticity in vCA1. Theranostics 15, 4808–4822. 10.7150/thno.106751 40225589 PMC11984413

[B9] Covarrubias A. J. Perrone R. Grozio A. Verdin E. (2021). NAD(+) metabolism and its roles in cellular processes during ageing. Nat. Rev. Mol. Cell Biol. 22, 119–141. 10.1038/s41580-020-00313-x 33353981 PMC7963035

[B10] Cryan J. F. O'Riordan K. J. Cowan C. S. M. Sandhu K. V. Bastiaanssen T. F. S. Boehme M. (2019). The microbiota-gut-brain axis. Physiol. Rev. 99, 1877–2013. 10.1152/physrev.00018.2018 31460832

[B11] Dicks L. M. T. (2022). Gut bacteria and neurotransmitters. Microorganisms 10, 1838. 10.3390/microorganisms10091838 36144440 PMC9504309

[B12] Doi H. Horie T. (2010). Salicylic acid-induced hepatotoxicity triggered by oxidative stress. Chem. Biol. Interact. 183, 363–368. 10.1016/j.cbi.2009.11.024 19948161

[B13] Duan Q. Wang Z. Lu T. Chen J. Wang X. (2006). Comparison of 6-hydroxylmelatonin or melatonin in protecting neurons against ischemia/reperfusion-mediated injury. J. Pineal Res. 41, 351–357. 10.1111/j.1600-079X.2006.00374.x 17014692

[B14] Duman R. S. Aghajanian G. K. Sanacora G. Krystal J. H. (2016). Synaptic plasticity and depression: new insights from stress and rapid-acting antidepressants. Nat. Med. 22, 238–249. 10.1038/nm.4050 26937618 PMC5405628

[B15] Ebokaiwe A. P. Obasi D. O. Obeten U. Onyemuche T. (2023). Rutin co-treatment prevented cognitive impairment/depression-like behavior and decreased IDO activation following 35 days of ethanol administration in male Wistar rats. Alcohol 106, 22–29. 10.1016/j.alcohol.2022.10.002 36306976

[B16] Foster J. A. McVey Neufeld K. A. (2013). Gut-brain axis: how the microbiome influences anxiety and depression. Trends Neurosci. 36, 305–312. 10.1016/j.tins.2013.01.005 23384445

[B17] Gao M. Wang J. Liu P. Tu H. Zhang R. Zhang Y. (2023). Gut microbiota composition in depressive disorder: a systematic review, meta-analysis, and meta-regression. Transl. Psychiatry 13, 379. 10.1038/s41398-023-02670-5 38065935 PMC10709466

[B18] Goda R. Otsuka T. Iwamoto A. Kawai M. Shibata S. Furuse M. (2015). Serotonin levels in the dorsal raphe nuclei of both chipmunks and mice are enhanced by long photoperiod, but brain dopamine level response to photoperiod is species-specific. Neurosci. Lett. 593, 95–100. 10.1016/j.neulet.2015.03.035 25797183

[B19] Gómez F. Graugés P. López-Calderón A. Armario A. (1999). Abnormalities of hypothalamic-pituitary-adrenal and hypothalamic-somatotrophic axes in Fawn-Hooded rats. Eur. J. Endocrinol. 141, 290–296. 10.1530/eje.0.1410290 10474128

[B20] Gross M. Pinhasov A. (2016). Chronic mild stress in submissive mice: marked polydipsia and social avoidance without hedonic deficit in the sucrose preference test. Behav. Brain Res. 298, 25–34. 10.1016/j.bbr.2015.10.049 26522843

[B21] Guan Y. Wang J. Wu X. Song L. Wang Y. Gong M. (2021). Quercetin reverses chronic unpredictable mild stress-induced depression-like behavior in vivo by involving nuclear factor-E2-related factor 2. Brain Res. 1772, 147661. 10.1016/j.brainres.2021.147661 34529966

[B22] Herrman H. Patel V. Kieling C. Berk M. Buchweitz C. Cuijpers P. (2022). Time for united action on depression: a Lancet-World Psychiatric Association Commission. Lancet 399, 957–1022. 10.1016/s0140-6736(21)02141-3 35180424

[B23] Higuchi Y. Soga T. Parhar I. S. (2017). Regulatory pathways of monoamine oxidase A during social stress. Front. Neurosci. 11, 604. 10.3389/fnins.2017.00604 29163009 PMC5671571

[B24] Hill-Burns E. M. Debelius J. W. Morton J. T. Wissemann W. T. Lewis M. R. Wallen Z. D. (2017). Parkinson’s disease and Parkinson’s disease medications have distinct signatures of the gut microbiome. Mov. Disord. 32, 739–749. 10.1002/mds.26942 28195358 PMC5469442

[B26] Jia Y. Wang Y. Wang Z. Zhang Z. Zhang J. Zhang J. (2024). Neuroprotective effects of total phenolics from Hemerocallis citrina Baroni leaves through the PI3K/AKT pathway. Front. Pharmacol. 15, 1370619. 10.3389/fphar.2024.1370619 39070797 PMC11272554

[B27] Jin Y. Sun L. H. Yang W. Cui R. J. Xu S. B. (2019). The role of BDNF in the neuroimmune axis regulation of mood disorders. Front. Neurol. 10, 515. 10.3389/fneur.2019.00515 31231295 PMC6559010

[B28] Jung S. J. Woo H. T. Cho S. Park K. Jeong S. Lee Y. J. (2017). Association between body size, weight change and depression: systematic review and meta-analysis. Br. J. Psychiatry 211, 14–21. 10.1192/bjp.bp.116.186726 28428339

[B29] Kelly J. R. Keane V. O. Cryan J. F. Clarke G. Dinan T. G. (2019). Mood and microbes: gut to brain communication in depression. Gastroenterol. Clin. North Am. 48, 389–405. 10.1016/j.gtc.2019.04.006 31383278

[B30] Lach G. Schellekens H. Dinan T. G. Cryan J. F. (2018). Anxiety, depression, and the microbiome: a role for gut peptides. Neurotherapeutics 15, 36–59. 10.1007/s13311-017-0585-0 29134359 PMC5794698

[B31] Layunta E. Buey B. Mesonero J. E. Latorre E. (2021). Crosstalk between intestinal serotonergic system and pattern recognition receptors on the microbiota-gut-brain axis. Front. Endocrinol. (Lausanne) 12, 748254. 10.3389/fendo.2021.748254 34819919 PMC8607755

[B32] Liang X. Q. Mai P. Y. Qin H. Li S. Ou W. J. Liang J. (2022). Integrated 16S rRNA sequencing and metabolomics analysis to investigate the antidepressant role of Yang-Xin-Jie-Yu decoction on microbe-gut-metabolite in chronic unpredictable mild stress-induced depression rat model. Front. Pharmacol. 13, 972351. 10.3389/fphar.2022.972351 36249818 PMC9565485

[B33] Liu D. Gharavi R. Pitta M. Gleichmann M. Mattson M. P. (2009). Nicotinamide prevents NAD+ depletion and protects neurons against excitotoxicity and cerebral ischemia: NAD+ consumption by SIRT1 may endanger energetically compromised neurons. Neuromolecular Med. 11, 28–42. 10.1007/s12017-009-8058-1 19288225 PMC2677622

[B34] Liu J. Ye T. Yang S. Zhong X. He W. Xu M. (2022). Antidepressant-like activity, active components and related mechanism of Hemerocallis citrina Baroni extracts. Front. Pharmacol. 13, 967670. 10.3389/fphar.2022.967670 36110538 PMC9469015

[B35] Liu L. Wu Z. Lu Y. Lu W. Su G. Zhou Z. (2024). Effects of phototherapy on biopterin, neopterin, tryptophan, and behavioral neuroinflammatory reaction in patients with post-stroke depression. Sci. Rep. 14, 18368. 10.1038/s41598-024-68799-5 39112627 PMC11306333

[B36] Ma H. Li C. Wang J. Zhang X. Li M. Zhang R. (2021). Amygdala-hippocampal innervation modulates stress-induced depressive-like behaviors through AMPA receptors. Proc. Natl. Acad. Sci. U. S. A. 118, e2019409118. 10.1073/pnas.2019409118 33526688 PMC8017726

[B37] Marin I. A. Goertz J. E. Ren T. Rich S. S. Onengut-Gumuscu S. Farber E. (2017). Microbiota alteration is associated with the development of stress-induced despair behavior. Sci. Rep. 7, 43859. 10.1038/srep43859 28266612 PMC5339726

[B38] Martinowich K. Lu B. (2008). Interaction between BDNF and serotonin: role in mood disorders. Neuropsychopharmacology 33, 73–83. 10.1038/sj.npp.1301571 17882234

[B39] Meilan X. Xiangyun T. Hui L. Jinglan Z. Yushan J. Xia Q. (2021). Neuroprotective effect of fucoidan by regulating gut-microbiota-brain axis in alcohol withdrawal mice. J. Funct. Foods 86, 104726. 10.1016/j.jff.2021.104726

[B25] Miura H. Ozaki N. Sawada M. Isobe K. Ohta T. Nagatsu T. (2008). A link between stress and depression: shifts in the balance between the kynurenine and serotonin pathways of tryptophan metabolism and the etiology and pathophysiology of depression. Stress Health, 24 (04), 271–280. 10.1080/10253890701754068 18465467

[B40] Nemeroff C. B. Widerlöv E. Bissette G. Walléus H. Karlsson I. Eklund K. (1984). Elevated concentrations of CSF corticotropin-releasing factor-like immunoreactivity in depressed patients. Science 226, 1342–1344. 10.1126/science.6334362 6334362

[B41] Qiao Y. Zhao J. Li C. Zhang M. Wei L. Zhang X. (2020). Effect of combined chronic predictable and unpredictable stress on depression-like symptoms in mice. Ann. Transl. Med. 8, 942. 10.21037/atm-20-5168 32953742 PMC7475446

[B42] Rinwa P. Kumar A. (2013). Quercetin suppress microglial neuroinflammatory response and induce antidepressent-like effect in olfactory bulbectomized rats. Neuroscience 255, 86–98. 10.1016/j.neuroscience.2013.09.044 24095694

[B43] Rominger A. Cumming P. Brendel M. Xiong G. Zach C. Karch S. (2015). Altered serotonin and dopamine transporter availabilities in brain of depressed patients upon treatment with escitalopram: a [123 I]β-CIT SPECT study. Eur. Neuropsychopharmacol. 25, 873–881. 10.1016/j.euroneuro.2014.12.010 25819144

[B44] Root D. H. Hoffman A. F. Good C. H. Zhang S. Gigante E. Lupica C. R. (2015). Norepinephrine activates dopamine D4 receptors in the rat lateral habenula. J. Neurosci. 35, 3460–3469. 10.1523/jneurosci.4525-13.2015 25716845 PMC4339355

[B45] Satomura E. Baba H. Nakano Y. Maeshima H. Suzuki T. Arai H. (2011). Correlations between brain-derived neurotrophic factor and clinical symptoms in medicated patients with major depression. J. Affect. Disord. 135, 332–335. 10.1016/j.jad.2011.06.041 21774990

[B46] Sharma H. R. Thakur M. K. (2015). Correlation of ERα/ERβ expression with dendritic and behavioural changes in CUMS mice. Physiol. Behav. 145, 71–83. 10.1016/j.physbeh.2015.03.041 25837835

[B47] Shen Z. Xu Y. Jiang X. Wang Z. Guo Y. Pan W. (2019). Avicularin relieves depressive-like behaviors induced by chronic unpredictable mild stress in mice. Med. Sci. Monit. 25, 2777–2784. 10.12659/msm.912401 30986204 PMC6482862

[B48] Stevens B. R. Goel R. Seungbum K. Richards E. M. Holbert R. C. Pepine C. J. (2018). Increased human intestinal barrier permeability plasma biomarkers zonulin and FABP2 correlated with plasma LPS and altered gut microbiome in anxiety or depression. Gut 67, 1555–1557. 10.1136/gutjnl-2017-314759 28814485 PMC5851874

[B49] Uezu E. (1998). Effects of Hemerocallis on sleep in mice. Psychiatry Clin. Neurosci. 52, 136–137. 10.1111/j.1440-1819.1998.tb00992.x 9628113

[B50] Viladomiu M. Hontecillas R. Yuan L. Lu P. Bassaganya-Riera J. (2013). Nutritional protective mechanisms against gut inflammation. J. Nutr. Biochem. 24, 929–939. 10.1016/j.jnutbio.2013.01.006 23541470 PMC3730123

[B51] Vollmer L. L. Schmeltzer S. N. Ahlbrand R. Sah R. (2015). A potential role for the acid-sensing T cell death associated gene-8 (TDAG8) receptor in depression-like behavior. Physiol. Behav. 150, 78–82. 10.1016/j.physbeh.2015.03.012 25770699 PMC4546899

[B52] Voltarelli V. A. Alves de Souza R. W. Miyauchi K. Hauser C. J. Otterbein L. E. (2023). Heme: the lord of the iron ring. Antioxidants (Basel) 12, 1074. 10.3390/antiox12051074 37237940 PMC10215292

[B53] Wang F. Liao Y. Liao T. Wan J. Chen G. Zhu N. (2023). Simultaneous determination of Niacin and nicotinamide in dendrobium by HPLC. Chin. Wild Plant Resour. 4, 23–27. 10.3969/j.issn.1006-9690.2023.04.005

[B54] Watanabe C. Oyanagi E. Aoki T. Hamada H. Kawashima M. Yamagata T. (2023). Antidepressant properties of voluntary exercise mediated by gut microbiota. Biosci. Biotechnol. Biochem. 87, 1407–1419. 10.1093/bbb/zbad115 37667506

[B55] Xie P. Chen L. Wang J. Wang X. Yang S. Zhu G. (2024). Polysaccharides from Polygonatum cyrtonema Hua prevent post-traumatic stress disorder behaviors in mice: mechanisms from the perspective of synaptic injury, oxidative stress, and neuroinflammation. J. Ethnopharmacol. 319, 117165. 10.1016/j.jep.2023.117165 37696440

[B56] Yi L. T. Li J. Li H. C. Zhou Y. Su B. F. Yang K. F. (2012). Ethanol extracts from Hemerocallis citrina attenuate the decreases of brain-derived neurotrophic factor, TrkB levels in rat induced by corticosterone administration. J. Ethnopharmacol. 144, 328–334. 10.1016/j.jep.2012.09.016 22995443

[B57] Yu M. Jia H. Zhou C. Yang Y. Zhao Y. Yang M. (2017). Variations in gut microbiota and fecal metabolic phenotype associated with depression by 16S rRNA gene sequencing and LC/MS-based metabolomics. J. Pharm. Biomed. Anal. 138, 231–239. 10.1016/j.jpba.2017.02.008 28219800

[B58] Yu M. Jia H. M. Qin L. L. Zou Z. M. (2022). Gut microbiota and gut tissue metabolites involved in development and prevention of depression. J. Affect. Disord. 297, 8–17. 10.1016/j.jad.2021.10.016 34666115

[B59] Yunes R. A. Poluektova E. U. Vasileva E. V. Odorskaya M. V. Marsova M. V. Kovalev G. I. (2020). A multi-strain potential probiotic formulation of GABA-producing Lactobacillus plantarum 90sk and Bifidobacterium adolescentis 150 with antidepressant effects. Probiotics Antimicrob. Proteins 12, 973–979. 10.1007/s12602-019-09601-1 31677091

[B60] Zeng J. Ji Y. Luan F. Hu J. Rui Y. Liu Y. (2022). Xiaoyaosan ethyl acetate fraction alleviates depression-like behaviors in CUMS mice by promoting hippocampal neurogenesis via modulating the IGF-1Rβ/PI3K/Akt signaling pathway. J. Ethnopharmacol. 288, 115005. 10.1016/j.jep.2022.115005 35051601

[B61] Zhang Y. Cichewicz R. H. Nair M. G. (2004). Lipid peroxidation inhibitory compounds from daylily (Hemerocallis fulva) leaves. Life Sci. 75, 753–763. 10.1016/j.lfs.2004.03.002 15172183

[B62] Zheng M. Liu C. Pan F. Shi D. Zhang Y. (2012). Antidepressant-like effect of hyperoside isolated from Apocynum venetum leaves: possible cellular mechanisms. Phytomedicine 19, 145–149. 10.1016/j.phymed.2011.06.029 21802268

[B63] Zheng S. Pan L. Hou J. Liao A. Hou Y. Yu G. (2022). The role of wheat embryo globulin nutrients in improving cognitive dysfunction in AD rats. Food Funct. 13, 9856–9867. 10.1039/d2fo00815g 36047913

