# Supplementary material for: Hemerocallis citrina Baroni leaf total phenol alleviates depressive-like behaviors via modulating “microbiota-gut-brain” axis in chronic unpredictable mild stress -induced rats
Source: Front Pharmacol. 2025 Sep 8;16:1642515. doi: 10.3389/fphar.2025.1642515 (PMC12451002; doi:10.3389/fphar.2025.1642515)
Supplement: Supplementary file 1 [file Table1.docx]

Table S1 Identification results of potential differential metabolites in serum

| No. | RT(min) | *m/z* | Index | Biomarkers | Formula | Adduct | VIP | HMDB | KEGG | Mode | CUMS/Control | CUMS_HLTP/CUMS | Pathway |
| --- | --- | --- | --- | --- | --- | --- | --- | --- | --- | --- | --- | --- | --- |
| 1 | 0.7874 | 58.0647 | MW0111340 | Trimethylamine N-oxide | C_3_H_9_NO | [M+H-H_2_O]^+^ | 1.437509133 | HMDB0000925 | C01104 | pos | down | up | - |
| 2 | 0.8044 | 118.0861 | MW0168837 | Betaine | C_5_H_11_NO_2_ | [M+H]^+^ | 1.426026853 | HMDB0000043 | C00719 | pos | down | up | k |
| 3 | 0.8044 | 70.0648 | MW0110995 | Diethanolamine | C_4_H_11_NO_2_ | [M+H-2H_2_O]^+^ | 1.281892713 | HMDB0004437 | C06772 | pos | down | up | - |
| 4 | 0.8044 | 74.0596 | MEDP0366 | N,N-Dimethylformamide | C_3_H_7_NO | [M+H]^+^ | 1.233752219 | HMDB0001888 | C03134 | pos | down | up | - |
| 5 | 0.8561 | 218.1383 | MW0055500 | Levocarnitine propionate | C_10_H_19_NO_4_ | [M+H]^+^ | 1.331144234 | HMDB0000824 | C03017 | pos | down | up | - |
| 6 | 1.2439 | 166.0173 | MW0126398 | Quinolinic acid | C_7_H_5_NO_4_ | [M-H]^-^ | 1.306126863 | HMDB0000232 | C03722 | neg | down | up | b |
| 7 | 1.2963 | 133.1046 | MW0169424 | Ornithine | C_5_H_12_N_2_O_2_ | [M+H]^+^ | 1.380218948 | HMDB0000214 | C00077 | pos | down | up | e,i,l |
| 8 | 1.302 | 134.106 | MW0141836 | Phenylpropanolamine | C_9_H_13_NO | [M+H-H_2_O]^+^ | 1.373830702 | HMDB0001942 | C02343 | pos | down | up | - |
| 9 | 1.5655 | 262.013 | MW0168109 | Dopaquinone | C_9_H_9_NO_4_ | [M-2H+3Na]^+^ | 1.41779061 | HMDB0001229 | C00822 | pos | up | down | n |
| 10 | 1.6353 | 124.0574 | MW0126293 | Pyrazinamide | C_5_H_5_N_3_O | [M+H]^+^ | 1.389313139 | HMDB0014483 | C01956 | pos | down | up | - |
| 11 | 1.6353 | 123.0551 | MW0169549 | Niacinamide | C_6_H_6_N_2_O | [M+H]^+^ | 1.373346746 | HMDB0001406 | C00153 | pos | down | up | b |
| 12 | 1.7115 | 153.065 | MEDL02165 | 1,4-Dihydro-1-methyl-4-oxo-3-pyridinecarboxamide | C_7_H_8_N_2_O_2_ | [M+H]^+^ | 1.464462334 | HMDB0004194 | C05843 | pos | down | up | b |
| 13 | 1.7288 | 154.0491 | MW0004669 | Aminosalicylic Acid | C_7_H_7_NO_3_ | [M+H]^+^ | 1.373063728 | HMDB0014378 | C02518 | pos | down | up | - |
| 14 | 1.9551 | 303.0703 | MW0114971 | Acetyl-N-formyl-5-methoxykynurenamine | C_13_H_16_N_2_O_4_ | [M+K]^+^ | 1.431243766 | HMDB0004259 | C05642 | pos | down | up | - |
| 15 | 1.9954 | 200.0178 | MW0120693 | 4-Pyridoxolactone | C_8_H_7_NO_3_ | [M+Cl]^-^ | 1.41764083 | HMDB0003454 | C00971 | neg | down | up | - |
| 16 | 2.0129 | 115.0402 | MW0105089 | alpha-Ketoisovaleric acid | C_5_H_8_O_3_ | [M-H]^-^ | 1.449336076 | HMDB0000019 | C00141 | neg | down | up | d,g,m |
| 17 | 2.4129 | 241.0584 | MW0121543 | 5-Hydroxytryptophan | C_11_H_12_N_2_O_3_ | [M+Na-2H]^-^ | 1.266837461 | HMDB0000472 | C01017 | neg | down | up | a |
| 18 | 2.4304 | 188.0026 | MW0004043 | 3-Hydroxyanthranilic acid | C_7_H_7_NO_3_ | [M+Cl]^-^ | 1.431635839 | HMDB0001476 | C00632 | neg | down | up | a |
| 19 | 2.5641 | 257.1012 | MW0105052 | 3-Hydroxymonoethylglycinexylidide | C_12_H_18_N_2_O_2_ | [M+Cl]^-^ | 1.464062219 | HMDB0060678 | C16572 | neg | down | up | - |
| 20 | 2.5866 | 322.0117 | MW0006527 | Chloramphenicol | C_11_H_12_C_l2_N_2_O_5_ | [M+Cl]^+^ | 1.41222735 | HMDB0014589 | C00918 | pos | up | down | - |
| 21 | 2.622 | 295.14 | MEDN0417 | (Rs)-Mevalonic Acid | C_6_H_12_O_4_ | [2M-H]^-^ | 1.374186394 | HMDB0000227 | C00418 | neg | down | up | f |
| 22 | 2.6608 | 285.0618 | MEDN0207 | 6-Hydroxymelatonin | C_13_H_16_N_2_O_3_ | [M+K-2H]^-^ | 1.459395788 | HMDB0004081 | C05643 | neg | down | up | a |
| 23 | 2.7774 | 351.0695 | MW0123679 | Dihydropteroate | C_14_H_14_N_6_O_3_ | [M+K-2H]^-^ | 1.439299308 | HMDB0001412 | C00921 | neg | down | up | h |
| 24 | 2.7947 | 211.0591 | MW0131370 | Desaminotyrosine | C_9_H_10_O_3_ | [M+HCOO]^-^ | 1.453156387 | HMDB0002199 | C01744 | neg | down | up | - |
| 25 | 2.7947 | 293.0621 | MW0127402 | Azathioprine | C_9_H_7_N_7_O_2_S | [M+NH_4_-2H]^-^ | 1.406674468 | HMDB0015128 | C06837 | neg | down | up | - |
| 26 | 2.8293 | 339.1662 | MW0000185 | Codeine | C_18_H_21_NO_3_ | [M-H+CH_3_CN]^-^ | 1.443000522 | HMDB0004995 | C06174 | neg | down | up | - |
| 27 | 2.8466 | 181.992 | MW0126499 | Saccharin | C_7_H_5_NO_3_S | [M-H]^-^ | 1.432653252 | HMDB0029723 | C12283 | neg | down | up | - |
| 28 | 2.8653 | 141.0538 | MEDP1832 | Ethyl maltol | C_7_H_8_O_3_ | [M+H]^+^ | 1.461930263 | HMDB0031735 | C20362 | pos | down | up | - |
| 29 | 2.8865 | 428.2479 | MW0169780 | Risperidone | C_23_H_27_FN_4_O_2_ | [M+NH_4_]^+^ | 1.432444446 | HMDB0005020 | C06861 | pos | down | up | - |
| 30 | 2.8946 | 144.0803 | MEDN0848 | Indole 3-Ethanol | C_10_H_11_NO | [M+H-H_2_O]^+^ | 1.411378305 | HMDB0003447 | C00955 | pos | down | up | - |
| 31 | 2.8985 | 280.0859 | MW0109195 | 4-Hydroxyphenylacetylglutamate | C_13_H_15_NO_6_ | [M-H]^-^ | 1.450954052 | HMDB0006061 | C05595 | neg | down | up | - |
| 32 | 3.166 | 106.0361 | MW0006140 | Benzaldehyde | C_7_H_6_O | [M+]^+^ | 1.424250189 | HMDB0006115 | C00261 | pos | down | up | - |
| 33 | 3.3505 | 299.0771 | MEDP0327 | N-γ-Acetyl-N-2-Formyl-5-Methoxykynurenamine | C_13_H_16_N_2_O_4_ | [M+Cl]^-^ | 1.464087422 | HMDB0004259 | C05642 | neg | down | up | a |
| 34 | 3.471 | 109.0295 | MW0006514 | Pyrocatechol | C_6_H_6_O_2_ | [M-H]^-^ | 1.452450516 | HMDB0000957 | C15571 | neg | down | up | - |
| 35 | 3.6356 | 847.4494 | MW0139584 | Rifabutin | C_46_H_62_N_4_O_11_ | [M+H]^+^ | 1.464875831 | HMDB0014753 | C07235 | pos | down | up | - |
| 36 | 3.6444 | 218.9693 | MW0123161 | Bromobenzene-2,3-oxide | C_6_H_5_BrO | [M+HCOO+2H]^+^ | 1.401648899 | HMDB0060446 | C14840 | pos | down | up | r |
| 37 | 3.8046 | 925.486 | MW0015886 | Araloside A | C_47_H_74_O_18_ | [M-H]^-^ | 1.269857804 | HMDB0034535 | C17540 | neg | down | up | - |
| 38 | 3.8644 | 159.103 | MW0014136 | 3-Hydroxyvalproic acid | C_8_H_16_O_3_ | [M-H]^-^ | 1.249963913 | HMDB0013899 | C16651 | neg | down | up | q |
| 39 | 3.9 | 247.0588 | MW0000277 | Harmine | C_13_H_12_N_2_O | [M+Cl]^-^ | 1.405174647 | HMDB0030311 | C06538 | neg | down | up | - |
| 40 | 4.0546 | 660.8609 | MW0115142 | Phytic acid | C_6_H_18_O_24_P_6_ | [M+H]^+^ | 1.44861032 | HMDB0003502 | C01204 | pos | down | up | j |
| 41 | 4.1773 | 343.0757 | MW0139601 | Rosmarinic acid | C_18_H_16_O_8_ | [M+H-H_2_O]^+^ | 1.437921144 | HMDB0003572 | C01850 | pos | down | up | - |
| 42 | 4.1911 | 461.1035 | MW0137862 | Daidzin | C_21_H_20_O_9_ | [M+HCOO]^-^ | 1.427984513 | HMDB0033991 | C10216 | neg | down | up | - |
| 43 | 4.3117 | 137.0247 | MW0009710 | Salicylic acid | C_7_H_6_O_3_ | [M-H]^-^ | 1.430193293 | HMDB0001895 | C00805 | neg | down | up | - |
| 44 | 4.6156 | 176.07 | MW0124410 | Indoleacetic acid | C_10_H_9_NO_2_ | [M+H]^+^ | 1.455288946 | HMDB0000197 | C00954 | pos | down | up | a |
| 45 | 5.0003 | 205.9795 | FDATN00199 | p-Toluenesulfonic acid | C_7_H_8_O_3_S | [M+Cl]^-^ | 1.429477504 | HMDB0059933 | C06677 | neg | down | up | - |
| 46 | 5.0503 | 116.0506 | MW0009416 | Benzeneacetonitrile | C_8_H_7_N | [M-H]^-^ | 1.425721242 | HMDB0034171 | C16074 | neg | down | up | - |
| 47 | 5.1021 | 335.2227 | MEDN0785 | LTB4 | C_20_H_32_O_4_ | [M-H]^-^ | 1.412586774 | HMDB0001085 | C02165 | neg | down | up | o |
| 48 | 5.2056 | 407.28 | MW0015712 | alpha-Muricholic acid | C_24_H_40_O_5_ | [M-H]^-^ | 1.47169895 | HMDB0000506 | C17647 | neg | up | down | - |
| 49 | 5.2056 | 571.2863 | MEDP1233 | D-Urobilinogen | C_33_H_42_N_4_O_6_ | [M-H_2_O-H]^-^ | 1.457675699 | HMDB0004158 | C05791 | neg | up | down | c |
| 50 | 5.4125 | 432.3089 | MW0053750 | Glycochenodeoxycholic acid | C_26_H_43_NO_5_ | [M+H-H_2_O]^+^ | 1.444151126 | HMDB0000637 | C05466 | pos | up | down | p |
| 51 | 5.43 | 465.3043 | MW0017085 | Cholesterol sulfate | C_27_H_46_O_4_S | [M-H]^-^ | 1.434389762 | HMDB0000653 | C18043 | neg | up | down | s |
| 52 | 5.551 | 245.0492 | MW0115129 | 3-Methyl-1-(2,4,6-trihydroxyphenyl)-1-butanone | C_11_H_14_O_4_ | [M+Cl]^-^ | 1.387288336 | HMDB0033798 | C07350 | neg | down | up | - |
| 53 | 5.551 | 327.0518 | MW0137751 | Coumestrol | C_15_H_8_O_5_ | [M+CH_3_COO]^-^ | 1.367938125 | HMDB0002326 | C10205 | neg | down | up | - |
| 54 | 5.6323 | 353.2469 | MW0054008 | Ethyl icosapentate | C_22_H_34_O_2_ | [M+Na]^+^ | 1.46066028 | HMDB0039530 | C16184 | pos | up | down | - |
| 55 | 5.6398 | 514.2372 | MW0009853 | Telmisartan | C_33_H_30_N_4_O_2_ | [M+]^+^ | 1.46866639 | HMDB0015101 | C07710 | pos | up | down | - |
| 56 | 5.7758 | 581.2516 | MEDP1045 | Biliverdin | C_33_H_34_N_4_O_6_ | [M-H]^-^ | 1.437988558 | HMDB0001008 | C00500 | neg | up | down | c |
| 57 | 5.9509 | 463.2692 | MW0015159 | 7alpha,12alpha-Dihydroxy-3-oxochol-4-en-24-oic Acid | C_24_H_36_O_5_ | [M+CH_3_COO]^-^ | 1.451751397 | HMDB0000447 | C15568 | neg | up | down | - |
| 58 | 5.9948 | 544.3035 | MW0145022 | Americine | C_31_H_39_N_5_O_4_ | [M-H]^-^ | 1.464416144 | HMDB0034441 | C09996 | neg | down | up | - |
| 59 | 6.0113 | 585.3351 | MW0108758 | Nelfinavir | C_32_H_45_N_3_O_4_S | [M+NH_4_]^+^ | 1.444837323 | HMDB0014365 | C07257 | pos | down | up | - |
| 60 | 6.0468 | 429.2602 | MW0055067 | Mifepristone | C_29_H_35_NO_2_ | [M+]^+^ | 1.46460542 | HMDB0014972 | C07652 | pos | up | down | - |
| 61 | 6.1411 | 392.2885 | FDATN01560 | Ursodeoxycholic acid | C_24_H_40_O_4_ | [M-]^-^ | 1.467238134 | HMDB0000946 | C07880 | neg | up | down | - |
| 62 | 6.5967 | 321.1687 | MW0015772 | Androst-5-ene-3,17-dione | C_19_H_26_O_2_ | [M+Cl]^-^ | 1.354249699 | HMDB0062415 | C20252 | neg | up | down | - |
| 63 | 7.733 | 377.2655 | MW0063480 | Sclareol | C_20_H_36_O_2_ | [M+Na+HCOOH]^+^ | 1.4225159 | HMDB0036827 | C09183 | pos | up | down | - |

(a: Tryptophan metabolism; b: Nicotinate and nicotinamide metabolism; c: Porphyrin metabolism; d: Valine biosynthesis; e: Arginine biosynthesis; f: Terpenoid backbone biosynthesis; g: Pantothenate and CoA biosynthesis; h: Folate biosynthesis; i: Glutathione metabolism; j: Inositol phosphate metabolism; k: Glycine metabolism; l: Arginine metabolism; m: Valine degradation; n: Tyrosine metabolism; o: Arachidonic acid metabolism; p: Primary bile acid biosynthesis; q: Drug metabolism - cytochrome P450; r: Metabolism of xenobiotics by cytochrome P450; s: Steroid hormone biosynthesis)
