# Supplementary material for: Hemerocallis citrina Baroni leaf total phenol alleviates depressive-like behaviors via modulating “microbiota-gut-brain” axis in chronic unpredictable mild stress -induced rats
Source: Front Pharmacol. 2025 Sep 8;16:1642515. doi: 10.3389/fphar.2025.1642515 (PMC12451002; doi:10.3389/fphar.2025.1642515)
Supplement: Supplementary file 2 [file Table2.docx]

Table S2 Analysis results of metabolic pathways for differential metabolites

| Pathway | Total | Hits | Raw p | -log(p) | Holm adjust | FDR | Impact |
| --- | --- | --- | --- | --- | --- | --- | --- |
| Tryptophan metabolism | 41 | 5 | 0.0002 | 3.7516 | 0.0142 | 0.0142 | 0.1825 |
| Nicotinate and nicotinamide metabolism | 15 | 3 | 0.0010 | 3.0148 | 0.0764 | 0.0387 | 0.1943 |
| Porphyrin metabolism | 31 | 2 | 0.0678 | 1.1685 | 1.0000 | 1.0000 | 0.0745 |
| Arginine biosynthesis | 14 | 1 | 0.1794 | 0.7461 | 1.0000 | 1.0000 | 0.0609 |
| Terpenoid backbone biosynthesis | 18 | 1 | 0.2248 | 0.6483 | 1.0000 | 1.0000 | 0.1143 |
| Glycine metabolism | 33 | 1 | 0.3744 | 0.4267 | 1.0000 | 1.0000 | 0.0515 |
| Arginine metabolism | 36 | 1 | 0.4008 | 0.3971 | 1.0000 | 1.0000 | 0.1640 |
| Valine degradation | 40 | 1 | 0.4344 | 0.3622 | 1.0000 | 1.0000 | 0.0108 |
| Arachidonic acid metabolism | 44 | 1 | 0.4661 | 0.3315 | 1.0000 | 1.0000 | 0.0291 |
| Primary bile acid biosynthesis | 46 | 1 | 0.4814 | 0.3175 | 1.0000 | 1.0000 | 0.0098 |
