# Supplementary material for: Hemerocallis citrina Baroni leaf total phenol alleviates depressive-like behaviors via modulating “microbiota-gut-brain” axis in chronic unpredictable mild stress -induced rats
Source: Front Pharmacol. 2025 Sep 8;16:1642515. doi: 10.3389/fphar.2025.1642515 (PMC12451002; doi:10.3389/fphar.2025.1642515)
Supplement: Supplementary file 3 [file DataSheet2.docx]

**
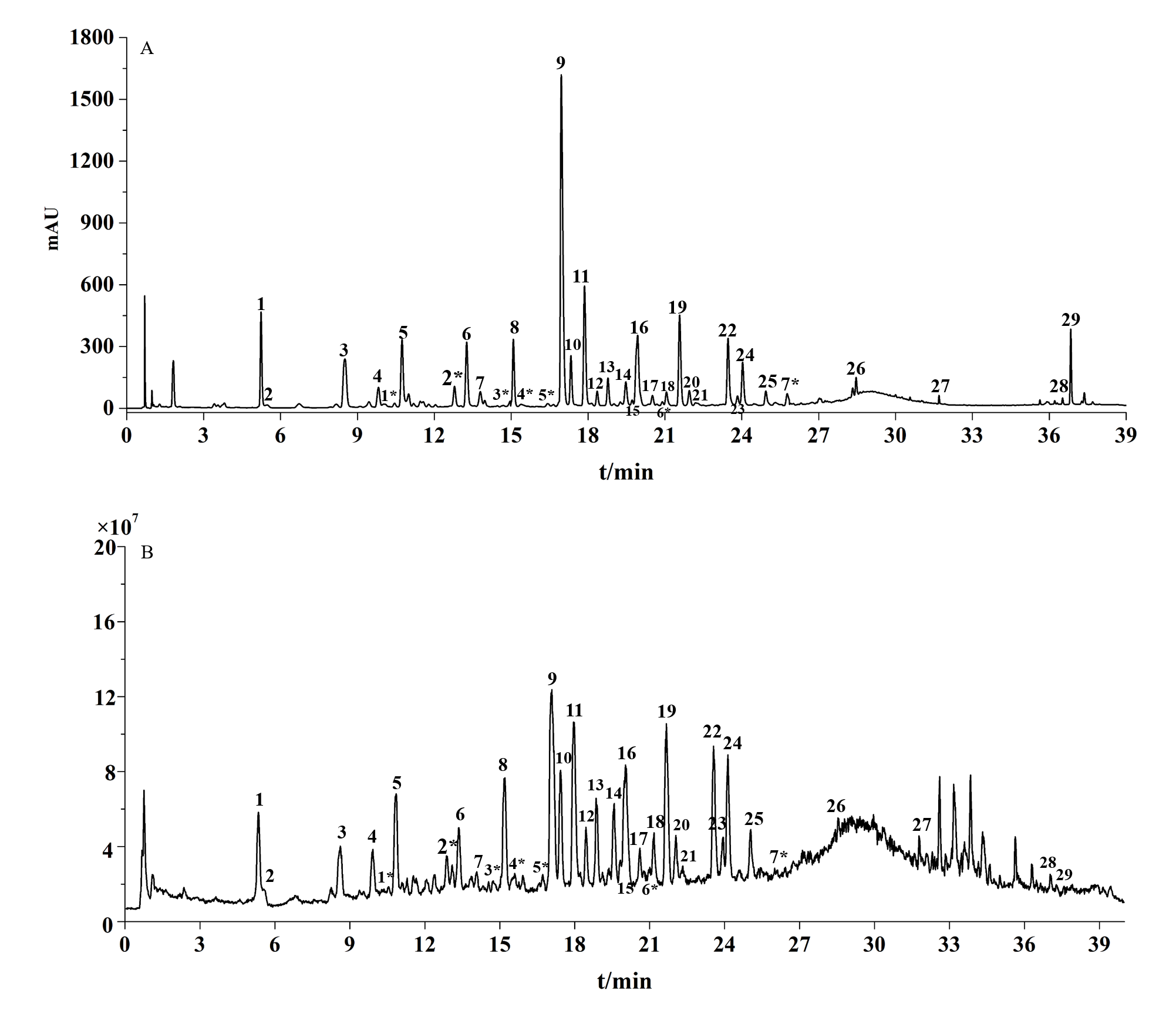
**

Fig. S1. UPLC-DAD chromatogram (A) and TIC in negative ion mode of HLTP (B).


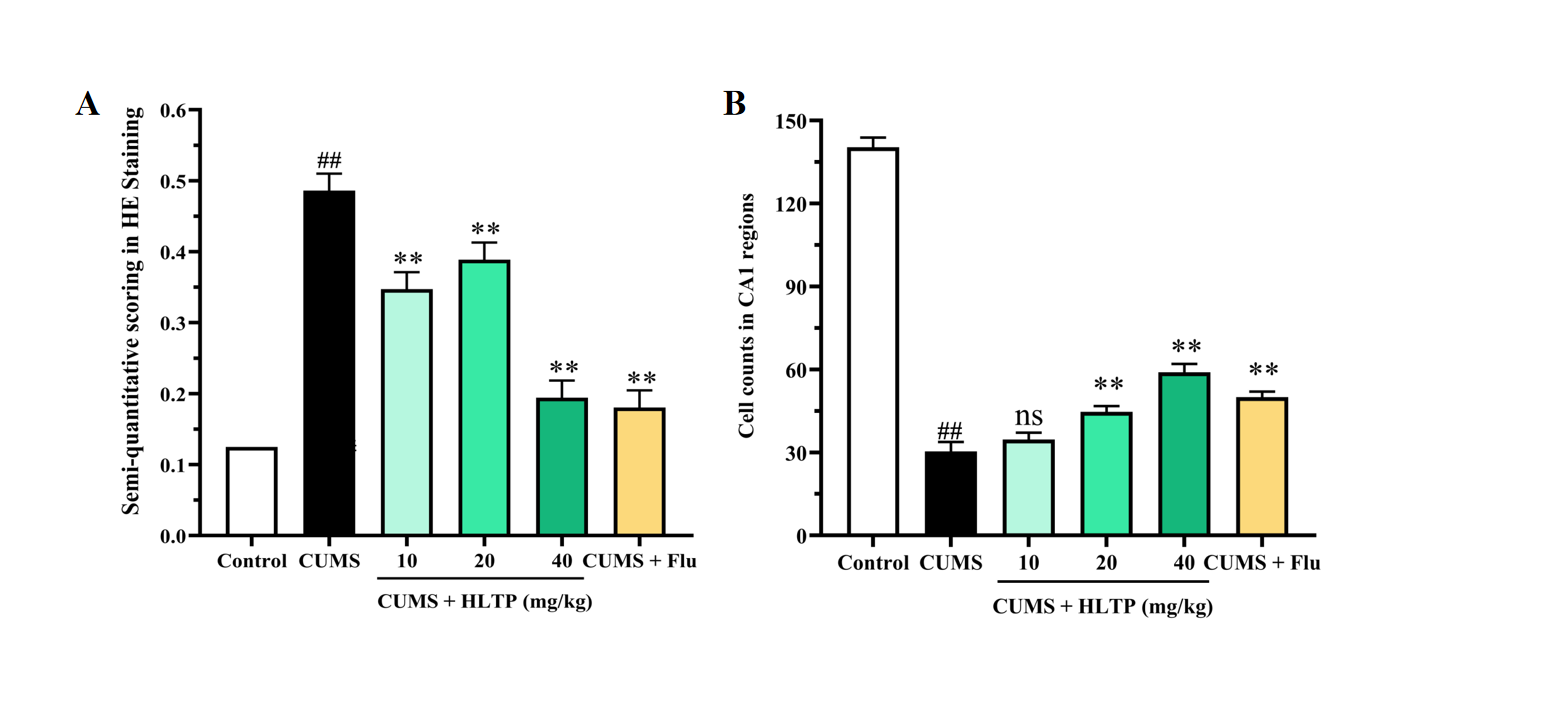


Fig. S2. Semi-quantitative analysis scores of H&E staining (A) and neuronal cell counts in the CA1 region from Nissl staining (B). ^##^*P* < 0.01 vs. Control; ***P* < 0.01 vs. CUMS. “ns” indicates no significant difference.
