# Supplementary material for: Hemerocallis citrina Baroni leaf total phenol alleviates depressive-like behaviors via modulating “microbiota-gut-brain” axis in chronic unpredictable mild stress -induced rats
Source: Front Pharmacol. 2025 Sep 8;16:1642515. doi: 10.3389/fphar.2025.1642515 (PMC12451002; doi:10.3389/fphar.2025.1642515)
Supplement: Supplementary file 4 [file DataSheet1.docx]

**supplementary materials-Presentation**

1. Behavioral tests

1.1 Sucrose preference test (SPT)

Two bottles (1% sucrose solution and tap water) were placed on each cage housing a single rat. After 2 days of adaptation with free access to both bottles (positions swapped every 6 h), rats underwent 24 h water/food deprivation. Subsequently, two identical bottles (1% sucrose solution vs. tap water) were provided for 2 h. Sucrose and water consumption were quantified by measuring pre- and post-test bottle weight differences. Sucrose preference (%) = [Sucrose consumption / (Sucrose + Tap water consumption)] × 100%.

1.2 Forced swimming test (FST)

Rats were placed in a transparent cylindrical glass container filled with water (depth: 50 cm) at ambient temperature for 6 min. The initial 2 min served as acclimation period, while immobility time (floating motionlessly with only necessary movements to keep the head above water) during the final 4 min was quantified.

1.3 Novelty suppressed feeding test (NSFT)

A wooden chamber (75×75×40 cm) with open top was used. Two to three food pellets were placed at the center of the chamber floor. After 24 h fasting, rats were placed in the chamber. The latency to feeding (time from placement to first bite) was recorded, with a maximum observation duration of 10 min.

1.4 Open field test (OFT)

Rats were individually placed in an open-field arena (75×75×40 cm) with black-painted walls and a floor divided into 16 (4×4) equal grids. Each rat was positioned at the center of the arena and allowed to freely explore for 5 min. The number of line crossings (complete grid transitions) and rearings (upright postures with both forelimbs off the floor) were recorded.

2. Serum metabolomics analysis

Samples were thawed from -80°C storage, mixed with 300 μL 20% acetonitrile-methanol internal standard extraction solution, vortexed for 3 min, and centrifuged. The supernatant was stored at -20°C for 30 min, recentrifuged, and the final supernatant was collected for analysis.

Chromatographic Conditions: Column: Waters ACQUITY Premier HSS T3 Column (1.8 µm, 2.1 mm × 100 mm); Mobile phase: A: 0.1% formic acid/water, B: 0.1% formic acid/acetonitrile; Column temperature: 40°C; Flow rate: 0.4 mL/min; Injection volume: 4 μL. Gradient elution program:0–2 min: 5% → 20% B; 2–5 min: 20% → 60% B; 5–6 min: 60% → 99% B; 6–7.5 min: 99% B; 7.5–7.6 min: 99% → 5% B; 7.6–10 min: 5% B;

Analysis was conducted using an AB TripleTOF 6600 mass spectrometer in both positive and negative ion modes. Parameters: acquisition time 10 min, nebulizer gas 50 psi, auxiliary heating gas 60 psi, curtain gas 35 psi, declustering voltage ±60 V, MS1 collision energy ±10 V, MS2 collision energy ±30 V.
